# The microbiome protects against septic hyperinflammation and bacterial proliferation in a zebrafish model of blood infection with *Escherichia coli* and mycobacteria

**DOI:** 10.3389/fimmu.2026.1837804

**Published:** 2026-07-02

**Authors:** Li Liu, Bjørn E. V. Koch, Elke H. J. Krekels, Herman P. Spaink

**Affiliations:** 1 Institute of Biology Leiden, Animal Science and Health, Leiden University, Leiden, Netherlands; 2 Leiden Academic Centre for Drug Research, Leiden University, Leiden, Netherlands; 3 Certara Inc., Princeton, NJ, United States

**Keywords:** *Escherichia coli*, microbiome, nontuberculous mycobacteria, TLR2, zebrafish

## Abstract

The microbiome is an important immune regulator, but the mechanisms by which commensal microbes shape systemic host defense during bloodstream infection remain poorly defined and commonly used pre-clinical models have practical, ethical and scientific limitations. Here, we establish a gnotobiotic zebrafish larval model to investigate microbiome-dependent protection against systemic blood infection by *Escherichia coli (E. coli)* bacteria, an important cause of early onset neonatal sepsis. We also use nontuberculous mycobacteria to infect zebrafish larvae to investigate the contribution of Toll-like receptor 2 (TLR2) in the defense responses. Germ-free (GF) and conventionalized (CONVD) larvae derived from the same clutches were systemically infected with *E. coli*, revealing that microbiome colonization significantly reduces early mortality. RNAseq revealed a conserved core immune activation program in both GF and CONVD larvae, but the absence of a microbiome was associated with a broader transcriptional response and stronger repression of metabolic pathways, suggesting that commensal microbes buffer infection-induced metabolic suppression. Extending this framework to nontuberculous mycobacteria, we performed systemic infections with fluorescent *Mycobacterium marinum* and *M. avium* in *tlr2* wild-type and mutant larvae under GF and CONVD conditions. While survival was largely unchanged, imaging-based quantification demonstrated increased bacterial proliferation in *tlr2* mutants and in GF larvae, with microbiome-mediated restriction of bacterial burden evident in wild-type but not *tlr2*-deficient hosts. Together, these data show that microbiome colonization buffers septic outcomes by reshaping systemic inflammatory and metabolic programs and identify TLR2 as a key node linking microbial colonization to effective host defense during nontuberculous mycobacterial infection.

## 1 Introduction

The microbiome has emerged as a central regulator of host immunity, influencing immune development, inflammatory tone, and metabolic homeostasis (1). Early-life microbial colonization is particularly important, as it shapes immune system maturation and calibrates host responsiveness to microbial stimuli. Germ-free animal models have for instance demonstrated that the absence of commensal microbes leads to profound defects in innate and adaptive immune responses, altered metabolism, and increased susceptibility to infection (2, 3), but it is unknown how this protective effect is achieved at a functional level. Many factors such as immunostimulatory effects and stimulation of anti-inflammatory signaling may be involved, but the complexity and dynamic nature of host-microbiome interactions makes it a challenging topic to address. Mouse models of sepsis have brought useful insights on the host-pathogen and host-microbiome interplay (4, 5), but it is difficult to integrate infectious challenges and microbiome alterations into pups, illustrating the need for new animal models.

Neonatal sepsis is among the most common causes of neonatal and infant death (6). It is classified either as early or late onset, depending on the time of manifestation. Furthermore, early and late onset differ in the most common causative pathogens with early onset sepsis being predominantly caused by *Streptococcus agalactiae* and *Escherichia coli* (*E. coli*) (7). The condition is treated with empirical antibiotics treatment but there is an urgent need for refinements to this approach requiring the identification of reliable biomarkers to guide treatment decisions (8, 9). A healthy microbiome confers significant protection against neonatal sepsis (10), suggesting that a preclinical model based on modification of the microbiome could offer valuable insight into the regulation of the immune response. In the future, such a model could be useful in the identification and testing of new treatment options as well.

Toll-like receptors (TLRs) play a pivotal role in sensing microbial products and orchestrating innate immune responses (11, 12). Among them, Toll-like receptor 2 (TLR2) recognizes a broad range of bacterial ligands, including lipoproteins and cell wall components from both Gram-positive and Gram-negative bacteria, as well as mycobacteria (13–15). TLR2 signaling has been implicated in inflammatory responses, metabolic regulation, and host defense against diverse pathogens (16–19). Importantly, accumulating evidence suggests that TLR2 does not function in isolation but instead integrates microbial cues derived from the commensal microbiota to fine-tune immune and metabolic programs (20–22). How TLR2-dependent signaling intersects with microbiome colonization to regulate host responses during systemic infection is poorly defined.

Zebrafish (*Danio rerio*) provide a powerful vertebrate model to investigate host-microbe interactions *in vivo* (23, 24). Their optical transparency, conserved innate immune system, and tractability for germ-free and gnotobiotic approaches make them particularly well suited for studying systemic infections and immune dynamics at the whole-organism level (23, 24). Zebrafish larval sepsis models based on challenge with lipopolysaccharide (LPS) (25) and live *E. coli* bacteria (26, 27) have already been developed and found to recapitulate many hallmarks of human sepsis.

We have conducted a series of experiments using zebrafish larvae to elucidate how the microbiome modulates immune reactions and inflammatory states (28, 29). Here we modify the existing sepsis infection models by introducing the microbiome as a variable, aiming to investigate how microbiome-immune crosstalk shapes infection outcomes. Our main objective is to determine whether the microbiome protects against septic hyperinflammation and bacterial proliferation, and to identify the molecular mechanisms underlying this protection, with a focus on TLR2 signaling. This work provides new insights into microbiome-immune crosstalk and suggests that modulation of microbiome-derived signals or TLR2-dependent pathways may represent promising strategies to enhance host resistance to bloodstream infections.

## 2 Materials and methods

### 2.1 Zebrafish maintenance and mutant line construction

All adult and larval zebrafish were maintained and all animal experiments described in this study were conducted in accordance with the EU Animal Protection Directive 2010/63/EU, under the supervision of the Animal Welfare Body of Leiden University. The culture of adult zebrafish was approved by the university’s local animal welfare committee (DEC; license number: protocol 14, 198). No adult zebrafish were sacrificed in this study. Adult fish were kept at 28 °C under a 14 h:10 h light-dark cycle and maintained according to standard protocols (http://zfin.org). Embryos and larvae were grown in laboratory-manufactured egg water (containing 60 mg/L instant ocean sea salts) at 28 °C with a 14 h:10 h light-dark cycle. All experiments involving bacterial injection and RNAseq analysis were performed on larvae up to 5 days post fertilization (dpf), prior to the onset of free feeding. According to the EU Animal Protection Directive 2010/63/EU, such early-stage larvae are not subject to animal experimentation regulations.

The *tlr2^sa19423^* mutant (further referred as *tlr2^-/-^* or *tlr2* mutant) line was obtained from the Sanger Institute Zebrafish Mutation Resource (Hinxton, Cambridge, UK) and shipped by the Zebrafish Resource Center of the Karlsruhe Institute of Technology. The line was identified by sequencing an ENU-mutagenized zebrafish library (ZFIN Cat# ZDB-ALT-131217-14694, RRID: ZFIN_ZDB-ALT-131217-1469) (30). All mutant alleles were confirmed by sequencing and the homozygote carriers of the mutations were outcrossed at least three times to wild-type fish (AB/TL strains) to minimize background mutations. Homozygote mutants and their wild-type siblings (further referred as *tlr2^+/+^*) were used to generate models in this study.

### 2.2 Bacterial inoculum preparation

Commercially available *Escherichia coli* (*ATCC 25922*) were chemically transformed with the plasmid pMP2463 (31), conferring gentamicin resistance. To prepare bacterial inoculum, cryopreserved stock was inoculated into liquid Luria-Bertani (LB) medium containing gentamicin (40 μg/mL) and cultured in an incubator at 37 °C with shaking (200 rpm) until the culture reached an OD600 of 0.5 ± 0.02 (approximately 12 hours). The bacteria were pelleted by gentle centrifugation at 4500 RCF for 3 minutes, washed with ice cold sterile PBS, spun down again and resuspended to a working OD600 of 5.0 in ice cold PBS. This concentrated working stock is used for microinjection, approximately 1 nL injection of this inoculum corresponds approximately to 800 CFU as assessed by colony counting from homogenized zebrafish embryos. By diluting or concentrating this working stock, we can prepare bacterial suspensions at various concentrations for injection. This bacterial inoculum was kept on ice until microinjection.

Two types of nontuberculous mycobacteria (NTM) strains, *Mycobacterium avium* subspecies *hominissuis Chester* (MAC 101, ATCC, 700898™) expressed mCherry fluorescent protein and *M. marinum* m20 (Mma20) expressed DsRed fluorescent protein, were used to infect zebrafish larvae in this study (32, 33). The MAC 101 mCherry strain was grown at 37 °C for 72 hours and the Mma20 DsRed strain was grown at 28 °C for 24 hours in Middlebrook 7H9 broth supplemented with 10% acid-albumin-dextrose-catalase (ADC) enrichment medium (Fort Richard, Auckland, New Zealand) and 50 µg/mL of hygromycin for selection of fluorescent. The bacteria were pelleted by gentle centrifugation at 4500 RCF for 3 minutes, washed with sterile PBS, spun down again and resuspended in sterile PBS containing 2% polyvinylpyrrolidone (PVP) 40 solution (Calbiochem, the Netherlands).

### 2.3 Germ-free larvae generation

Germ-free (GF) and conventionalized (CONVD) zebrafish embryos were generated following the previously described “Natural breeding method” with several modifications (28, 34). Briefly, freshly collected embryos were divided into two groups. The egg water used for all groups contained 60 mg/L Instant Ocean salts (Spectrum Brands, Blacksburg, USA). One group was treated with an antibiotic mix containing Ampicillin (250 µg/mL), Kanamycin (5 µg/mL) and amphotericin B (250 ng/mL) after the harvest of embryos and maintained in autoclaved sterile egg water. At 6 hours post fertilization (hpf), embryos from this group were surface-sterilized using 0.2% PVP-I and 0.003% bleach and subsequently rinsed with sterile egg water. At 3 dpf, half of the sterilized larvae were randomly transferred into egg water containing water collected from the conventionally reared petri dishes to reintroduce microbial exposure, forming the CONVD group. The remaining sterilized larvae were kept in sterile egg water as the GF group. Only morphologically normal larvae were selected for subsequent experiments. Sterile conditions in GF groups were monitored by daily plating 2  mL water from the GF group petri dish on tryptic soy agar and LB agar plates under aerobic conditions at 28 °C for 2 days.

### 2.4 Systemic bacterial microinjections

Systemic bacterial infections were established by microinjection into the duct of Cuvier, following previously described protocols (35). At 3 dpf, GF and CONVD larvae from AB/TL line were generated as described previously. Bacterial microinjections were performed 6 hours after CONVD establishment, a time point that balances adequate microbial conditioning with technical feasibility for microinjection, given that older larvae are increasingly difficult to inject reliably. Larvae were anaesthetized in 0.02% tricaine (3-aminobenzoic acid ethyl ester; Sigma-Aldrich, Zwijndrecht, Netherlands) sterilized egg water and aligned on 1% agarose coated petri dishes. *E. coli* bacterial suspensions were prepared at defined optical densities. A constant injection volume of approximately 1 nL per larva was used for all microinjections. A series of doses of suspension (500, 1000 and 4000 CFU) was achieved by adjusting the concentration of the bacterial suspension accordingly. Each dose was injected into the duct of Cuvier using a microinjector (FemtoJet 4i, Eppendorf, Hamburg, Germany) equipped with fine-tipped glass capillaries. Successful injection was confirmed by transient visualization of bacterial suspension entering the circulatory system. MAC 101 mCherry strain (1000, 2500 and 5000 CFU) and Mma20 DsRed strain (250, 500 and 500 CFU) were injected into the duct of Cuvier using the same method. For mock-injected controls, the same volume of sterile PBS was injected into the duct of Cuvier following identical procedures. For *E. coli* microinjections in AB/TL larvae, a total of 25 successfully injected larvae for each treatment group were included for the following analysis. For MAC 101 and Mma20 microinjections in AB/TL larvae, 25 injected larvae for each group were analyzed. For MAC 101 and Mma20 microinjections in *tlr2* wild-type or mutant larvae, 30 injected larvae for each group were analyzed. Data were collected from three independent experiments.

After injection, GF larvae were gently released into fresh sterile egg water and CONVD larvae were put back to the normal egg water and allowed to recover from the anesthesia before further incubation. Only larvae showing normal morphology and circulation after recovery were included in subsequent experiments. Survival rates were checked and recorded at 20, 40 and 48 hours post injection (hpi).

### 2.5 RNAseq processing and analysis

To perform the deep sequencing of larvae after systemic *E.coli* injection, samples from four groups (*E.coli* injected GF larvae, PBS injected GF larvae, *E.coli* injected CONVD larvae, PBS injected CONVD larvae) were collected at 4 h post infection (4 hpi). To determine the optimal time point for transcriptomic analysis, a preliminary time-course experiment was performed. At 2, 4, and 18 hpi, 8 larvae per *E.coli* injected group were homogenized in 200 µl sterile PBS, and solutions were spotted on LB media for CFU enumeration. Based on the observation that the difference in bacterial burden between GF and CONVD larvae was most pronounced at 4 hpi (**Supplementary Figure 1**), this time point was selected for subsequent RNAseq analysis. The extraction of total RNA from larvae of each group was using TRIzol Reagent (Life Technologies, Carlsbad, USA) according to the manufacturer’s instructions. To remove the DNA contamination, DNase treatment was conducted by using the DNase I Kit (Thermo Scientific, Waltham, USA). RNA sequencing of larvae from each group was conducted by GenomeScan B.V. (Leiden, The Netherlands) as previously described (30). Sequencing data of three biological replicates for each group were aligned and mapped to the zebrafish genome GRCz11 using Salmon v1.2.1 (36). Differential gene expression was analyzed by using DESeq2 v1.24.0 (37). Statistical significance was determined by a padj value of ≤ 0.05 (38). Gene Ontology (GO) term and Kyoto Encyclopedia of Genes and Genomes (KEGG) pathway enrichment analysis was performed in DAVID Bioinformatics Resources 6.8 (https://davidbioinformatics.nih.gov/).

### 2.6 Confocal imaging and quantification

For MAC 101 and Mma20 injection in *tlr2^+/+^* and *tlr2^-/-^* larvae under both GF and CONVD conditions, a total of 30 successfully injected larvae for each treatment group were included for the following analysis. After 48 hpi, all survived larvae were collected and imaged using the Leica Stellaris 5 confocal laser scanning microscope (Leica Microsystems, Wetzlar, Germany). The bacterial burden of the full body, anterior and posterior part of the body for the larvae from four experimental groups (GF *tlr2* wild-type group, GF *tlr2* mutant group, CONVD *tlr2* wild-type group and CONVD *tlr2* mutant group) were quantified using ImageJ software. The anterior and posterior parts of the body were defined using the end of the gut as the anatomical boundary. The bacterial burden were quantified using ImageJ software.

### 2.7 Statistics analysis

The statistical analysis was performed using the Graphpad Prism software (Version 10; GraphPad Software, San Diego, CA, USA). All experimental data in this study are shown as mean ± the standard deviation (SD). D’Agostino-Pearson and Shapiro-Wilk normality test was performed to determine the Gaussian distribution of the data. The statistical significant differences between groups was determined using one-way analysis of variance (ANOVA) and the Tukey-Kramer method was used for *post hoc* analysis. The significance was established as * *P* < 0.05, ** *P* < 0.01, *** *P*  <  0.001 and ****, *P* < 0.0001.

## 3 Results

### 3.1 Microbiome colonization confers protection against early mortality caused by *E. coli* infection in zebrafish embryos

**Figure 1:**
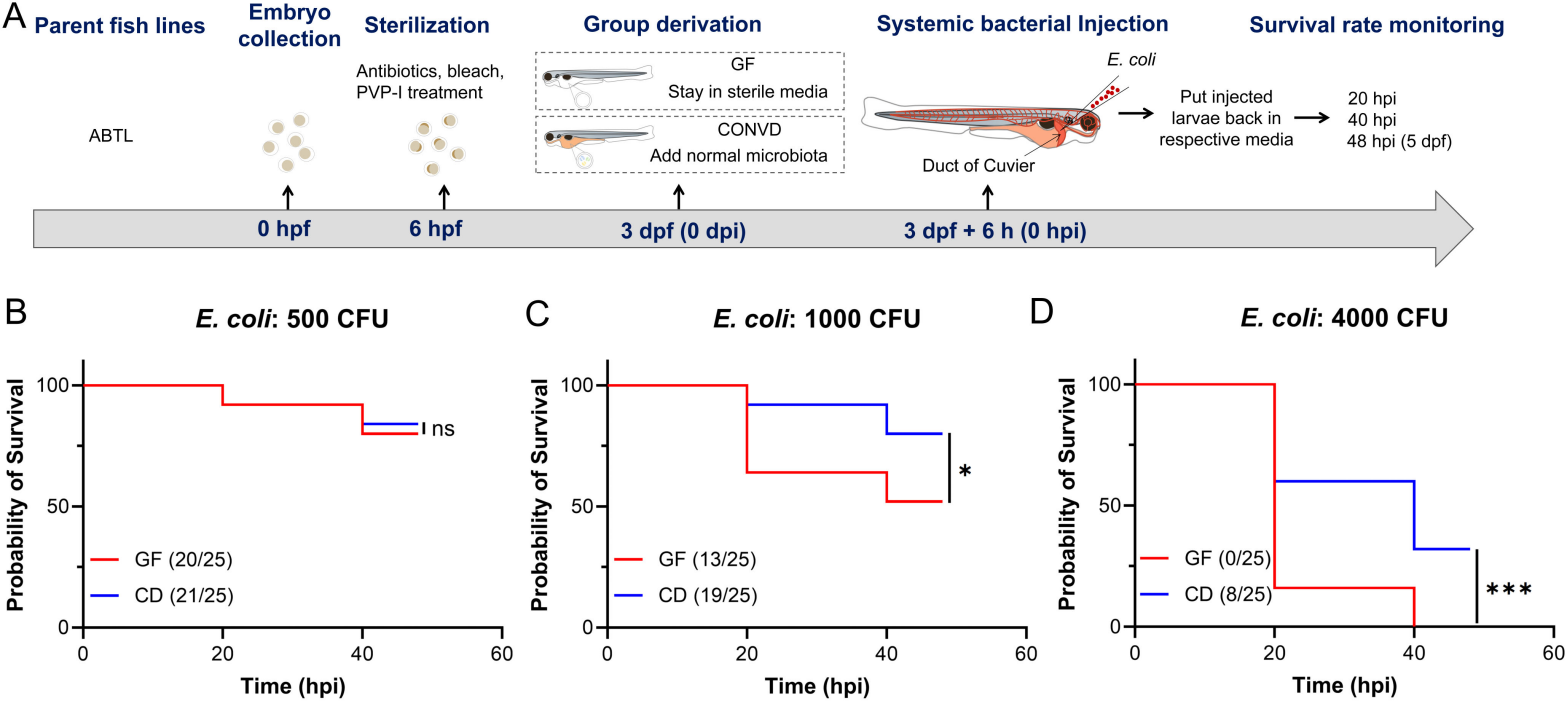
Probability of survival curves for zebrafish larvae infected with a series of doses of *E. coli* under the germ-free or conventionalized condition. **(A)** Schematic overview of the experimental workflow. Embryos were collected at 0 hour post fertilization (hpf). At 6 hpf, the exterior chorion membranes of embryos were sterilized through chemical treatment and embryos were maintained under germ-free (GF) conditions until 3 days post fertilization (dpf). At 3 dpf, when larvae had shed the chorion and developed open mouths and gastrointestinal tracts, the conventionalized (CONVD) group was generated by introducing microbes from the conventionally reared group. *E. coli* bacterial systemic microinjections were performed 6 hours after group division to allow stabilization of microbial conditions prior to infection. The larvae from GF and CONVD group were systemically infected with *E. coli* bacteri*a* via microinjection into the duct of Cuvier. A total of 25 successfully injected larvae for each treatment group were included for the following analysis. Survival rate monitoring was performed at 20, 40 and 48 hours post injection (hpi). **(B, D)** Kaplan Meier plots of larval survival rates after infection with increasing infectious burdens of *E. coli*, 500 CFU **(B)**, 1000 CFU **(C)** and 4000 CFU **(D)**. Data were collected from three independent experiments. Kaplan-Meier survival curve represented pooled data from all three experiments. Statistical curve comparisons by Mantel-Cox test. *P < 0.05, ***P < 0.001, ns, non-significant.

To assess the protective role of the microbiome against septic infection, GF and CONVD zebrafish larvae were infected with varying doses of *E. coli* (ATCC 25922), a reference strain of *E. coli* originating from a clinical isolate, and survival was monitored over time (**Figure 1A**). The results showed that under the low infectious burden (500 CFU) there was no significant difference in survival rate between GF group and CONVD group (**Figure 1B**). However, at higher infectious burdens (1000 and 4000 CFU), the presence of a microbiome conferred significant protection against early mortality, as the survival rate of larvae of the CONVD group was higher than the GF group (**Figures 1C, D**). Specifically, infection with 1000 CFU resulted in 76% survival rate in the CONVD group compared to 52% in the GF group at 48 hpi (**Figure 1C**). Infection with 4000 CFU resulted in 32% survival rate for the CONVD group, whereas no larvae survived in the GF group at 48 hpi (**Figure 1D**). Notably, the CONVD group, which at the time of infection had only been colonized for 6 hours, exhibited a reduced mortality compared to the GF group, indicating that even a relatively brief exposure to commensal microbes conferred some protection. These findings demonstrate that microbiome colonization provides early-life protection against systemic *E. coli* infection, highlighting the importance of microbial priming in shaping host defense capacity.

### 3.2 Transcriptomic alterations caused by *E. coli* infection differ in the presence or absence of a microbiome

**Figure 2:**
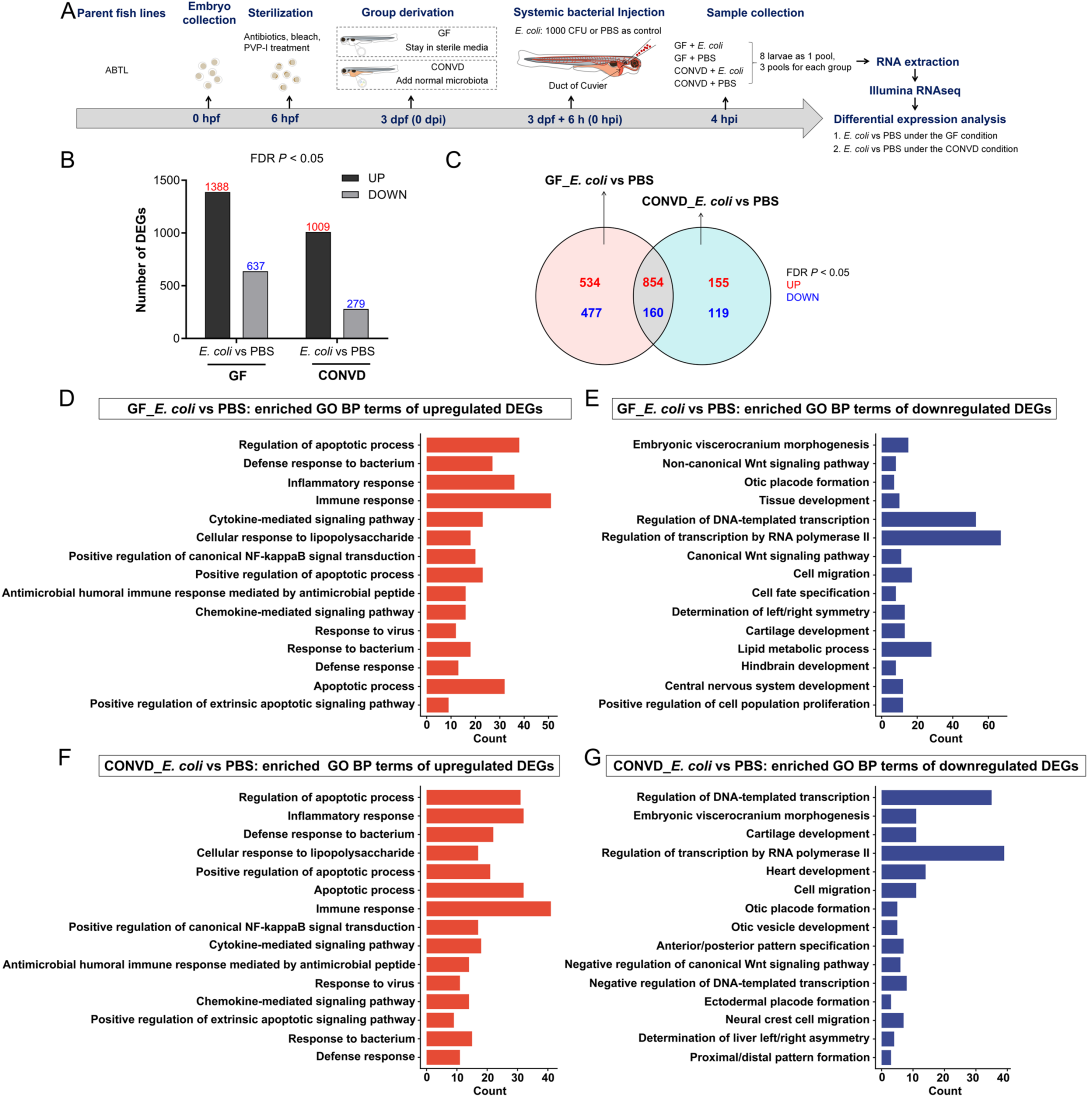
Differential gene expression profiles resulting from systemic infection with *E. coli* under the germ-free or conventionalized condition. **(A)** Schematic overview of the experimental workflow. At 3 days post fertilization (dpf), larvae were maintained as germ-free (GF) or conventionalized (CONVD) by exposure to a defined microbial community. *E. coli* bacterial systemic microinjections were performed 6 hours after group division to allow stabilization of microbial conditions prior to infection and injected larvae were collected at 4 hours post injection (hpi) for RNA extraction and Illumina RNA sequencing (8 larvae as 1 pool, 3 pools for each group). Differential expression analysis was performed for two comparisons: (i) *E. coli* injected larvae versus PBS control injected larvae under the GF condition, (ii) *E. coli* injected larvae versus PBS control injected larvae under the CONVD condition. **(B)** Numbers of differentially expressed genes (DEGs) in *E. coli* injected larvae versus PBS control injected larvae under GF and CONVD conditions. DEGs were assessed by *P* value < 0.05, which were adjusted using the Benjamini and Hochbery FDR correction (FDR *P* value < 0.05). Upregulated gene sets are shown in red and downregulated gene sets are shown in blue. **(C)** Venn diagram showing the overlap of DEGs identified in GF and CONVD larvae following *E. coli* systemic infection. Shared and condition-specific upregulated (red) and downregulated (blue) DEGs are indicated. **(D, E)** Top 15 gene ontology (GO) biological process (BP) term enrichment analysis of upregulated DEGs **(D)** and downregulated DEGs **(E)** under the GF condition. **(F, G)** Top 15 gene Ontology (GO) biological process (BP) term enrichment analysis of upregulated DEGs **(F)** and downregulated DEGs **(G)** under the CONVD condition.

In order to investigate the phenomenon of microbiome derived protection from lethality more closely, we performed RNAseq studies to compare the transcriptomic alterations caused by *E. coli* infection under GF and CONVD conditions (**Figure 2A**). In the GF condition, a signature set of 2025 significantly differentially expressed genes (DEGs) were identified in the *E. coli* infected larvae compared to PBS mock injection controls (FDR *P* value < 0.05), comprising 1388 upregulated and 637 downregulated genes (**Figure 2B**). In the CONVD condition, a signature set of 1288 DEGs were detected in the *E. coli* infected larvae compared to PBS mock injection controls (FDR *P* value < 0.05), including 1009 upregulated genes and 279 downregulated genes (**Figure 2B**). Among these, 854 upregulated and 160 downregulated DEGs were shared between the CONVD and GF conditions, indicating a core transcriptional program triggered by *E. coli* infection that is regulated in the same way irrespective of microbiome colonization status (**Figure 2C**). Moreover, the total number of DEGs was notably higher in the absence of the microbiome compared to the colonized condition, suggesting that microbiome colonization narrows and dampens the host transcriptional response to systemic infection. This pattern is consistent with the idea that commensal microbes precondition the host immune system, limiting excessive or dysregulated responses to bacterial sepsis.

For gene enrichment analysis of DEG signature sets identified above, Gene Ontology (GO) biological process (BP) term enrichment analysis was performed using the DAVID bioinformatics program. In summary, for the upregulated DEGs, the enriched pathways were similar under both GF and CONVD conditions and were dominated by immune response related processes (**Figures 2D, F**). In contrast, the downregulated DEGs showed microbiome colonized condition-specific enrichment patterns. For example, the lipid metabolic process pathway was enriched only among downregulated genes under the GF condition (**Figures 2E, G**), indicating that systemic *E. coli* infection more strongly suppresses host lipid metabolism in the absence of a microbiome. These results demonstrate that, while a core immune activation program is shared between GF and CONVD larvae, microbiome colonization reshapes the metabolic arm of the host response, buffering infection-induced repression of lipid metabolic pathways.

**Figure 3:**
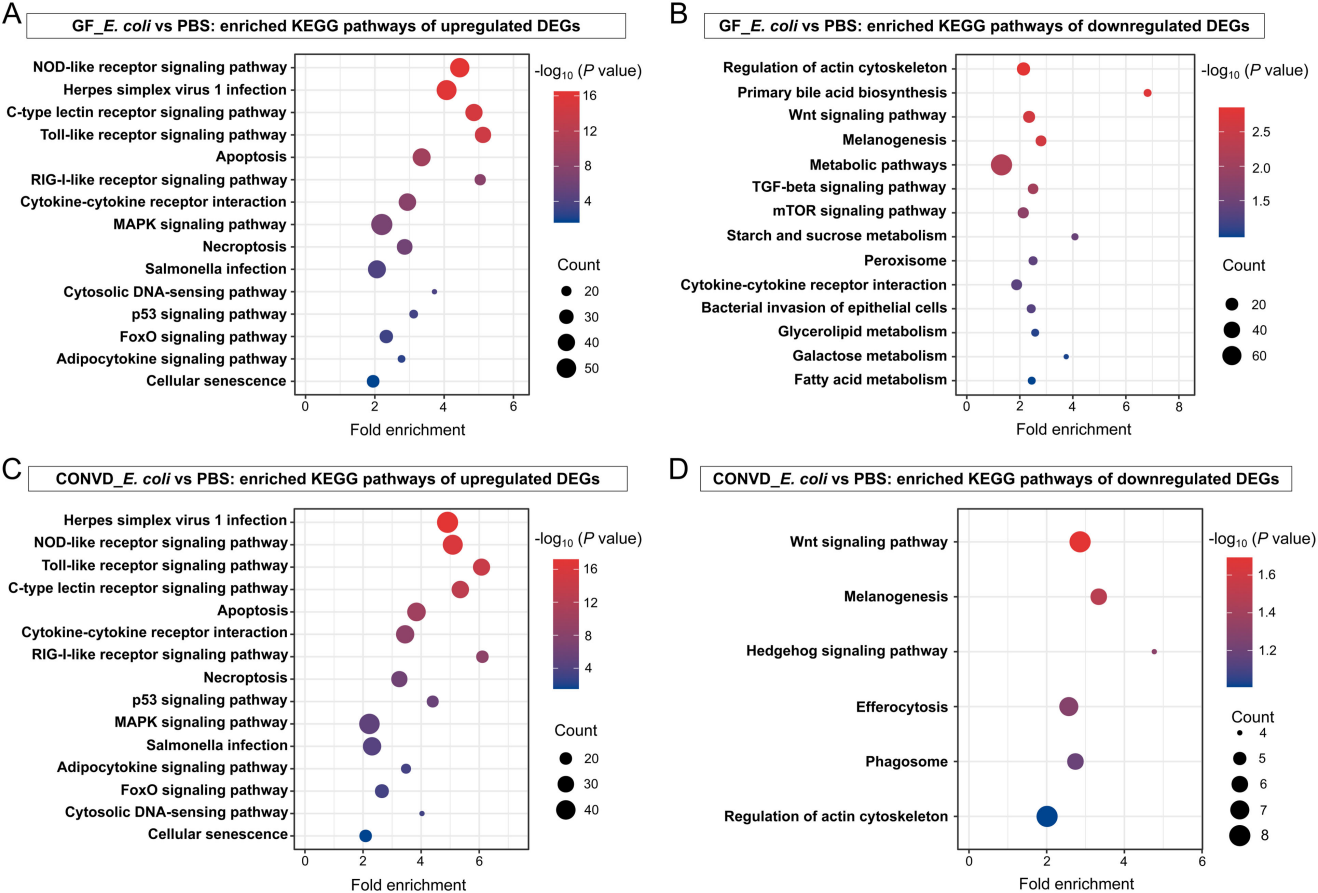
KEGG enriched pathway analysis of differentially expressed genes resulting from systemic infection with *E. coli* under the germ-free or conventionalized condition. **(A, B)** KEGG pathway enrichment analysis of upregulated DEGs **(A)** and downregulated DEGs **(B)** under the GF condition. **(C, D)** KEGG pathway enrichment analysis of upregulated DEGs **(C)** and downregulated DEGs **(D)** under the CONVD condition. KEGG pathway terms for DEGs were determined by using the hypergeometric test/Fisher’s exact test, with a threshold of *P* value < 0.05, which were adjusted using the Benjamini and Hochbery FDR correction.

To complement the GO analysis, we performed KEGG enrichment analysis on the same DEG sets. Across both GF and CONVD conditions, *E. coli* infection-induced upregulated DEGs were strongly enriched for immune-related pathways, including cytokine-mediated signaling, pattern recognition receptor signaling pathways, apoptosis and necroptosis (**Figures 3A, C**). These shared enrichments indicate the presence of a conserved core immune activation program triggered by *E. coli* infection, largely independent of microbiome colonization status. In contrast, downregulated DEGs exhibited marked differences between GF and CONVD larvae (**Figures 3B, D**). In GF larvae, downregulated genes were preferentially enriched for metabolic processes, including general metabolic processes, starch and sucrose metabolism, glycerolipid metabolism, galactose metabolism, and fatty acid metabolism, suggesting that systemic infection in the absence of a microbiome is accompanied by a broad repression of host metabolic functions (**Figure 3B**). Notably, these metabolic pathways were far less prominently enriched among downregulated DEGs in CONVD larvae, indicating that microbiome colonization buffers infection-induced suppression of metabolic gene programs (**Figure 3D**).

### 3.3 Microbiome-dependent modulation of immune and metabolic pathways during systemic *E. coli* infection

**Figure 4:**
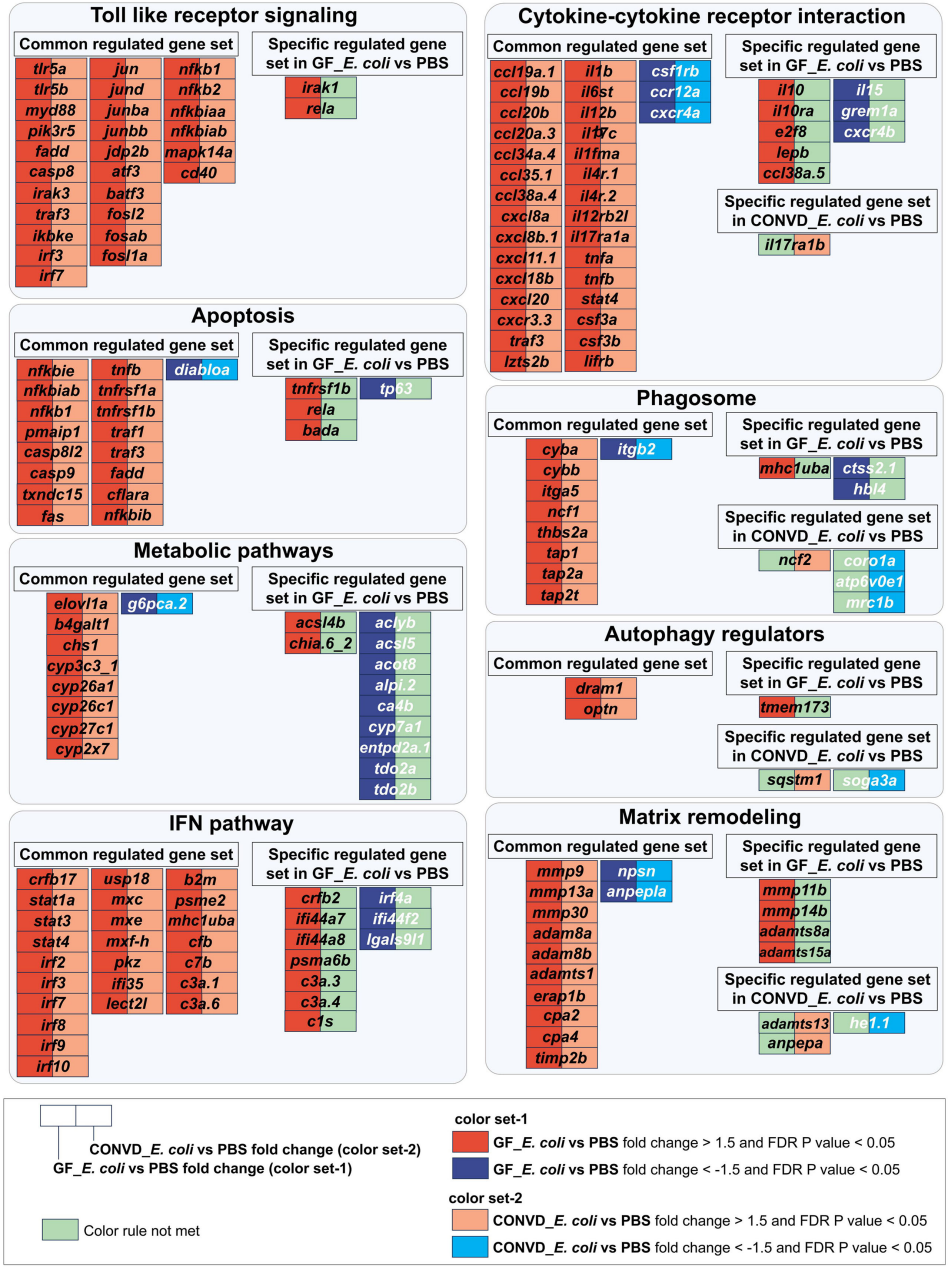
Common and specific gene expression profiles resulting from systemic infection with *E. coli* under the germ-free or conventionalized condition. In the visualization, the gene expression in the comparison of the *E. coli* injected larvae versus PBS control injected larvae under germ-free (GF) or conventionalized (CONVD) condition are depicted by color (deep orange, upregulated genes under the GF condition; deep blue, downregulated genes under the GF condition; light orange, upregulated genes under the CONVD condition; light blue, downregulated genes under the CONVD condition. FDR *P* value < 0.05).

To further resolve how microbiome colonization modulates host responses to systemic *E. coli* infection, we compared DEGs between infected and PBS-injected larvae under GF and CONVD conditions, focusing on key immune related and metabolic pathways (**Figure 4**). For each pathway, DEGs were classified into commonly regulated genes shared between GF and CONVD larvae, or condition-specific genes uniquely regulated in one condition. The details of the DEGs from the representative pathways under GF conditions are shown in **Supplementary Table 1**, while those under CONVD conditions are shown in **Supplementary Table 2**.

Across multiple immune-related pathways, including Toll-like receptor signaling, cytokine-cytokine receptor interaction, apoptosis, and the interferon (IFN) pathway, a substantial core set of genes was commonly regulated under both GF and CONVD conditions. These shared genes included canonical innate immune regulators such as myeloid differentiation primary response 88 (*myd88*), interleukin-1 receptor-associated kinase 3 (*irak3*), nuclear factor kappa-light-chain-enhancer of activated B cells (*nfkb*) family members, signal transducer and activator of transcription (*stat*) genes, transcription factors (AP-1 genes), interferon regulatory factors (*irf* genes), matrix metalloproteinase (*mmp*) family members, and chemokines (*cxcl* and *ccl* family members), indicating a conserved transcriptional program triggered by *E. coli* infection irrespective of microbiome status (**Figure 4**).

In contrast, condition-specific transcriptional signatures were evident across several pathways. In GF larvae, *E. coli* infection uniquely regulated genes involved in cytokine signaling (e.g. *il10*, *il10ra*), apoptosis (e.g. *tnfrsf1b*, *tp63*), and metabolic pathways, suggesting an exaggerated or dysregulated host response in the absence of microbial colonization. Notably, several complement components (e.g. *c3a.3*, *c1s*) were also preferentially induced under GF conditions, consistent with heightened inflammatory signaling. Conversely, CONVD larvae displayed a more restricted set of condition-specific genes, including selective regulation of cytokine receptors (*il17ra1b*), phagosome-associated genes (*ncf2*, *mrc1*b) and autophagy-related genes (*sqstm1*, *soga3a*). These findings suggest that microbiome colonization fine-tunes immune and tissue remodeling responses, potentially limiting excessive inflammation while preserving antimicrobial defense.

### 3.4 Absence of TLR2 as well as the microbiome promotes mycobacterial proliferation after systemic infection

**Figure 5:**
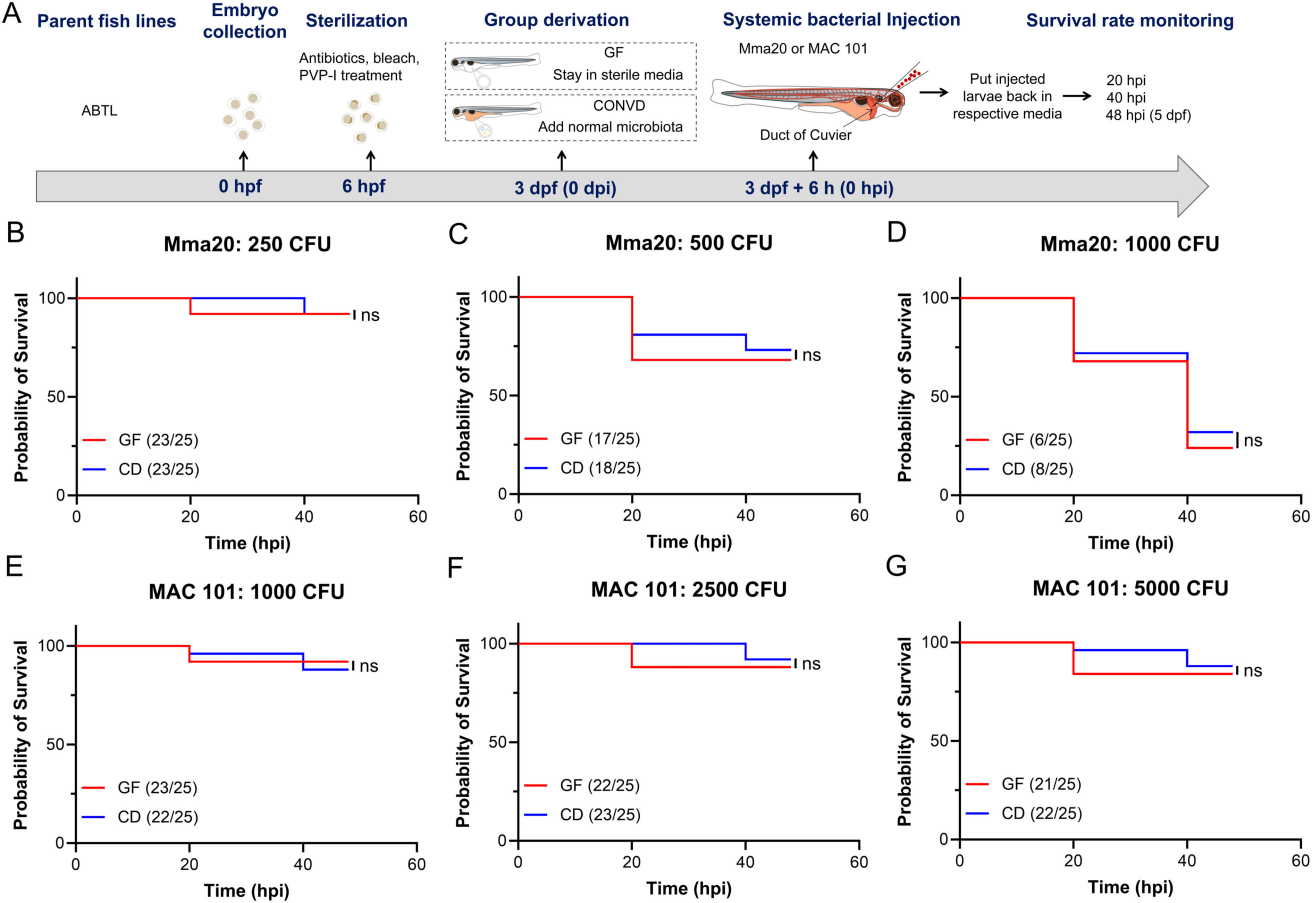
Probability of survival curves for zebrafish larvae infected with a series of doses of *M. marinum* Mma20 or *M. avium* MAC 101 under the germ-free or conventionalized condition. **(A)** Schematic overview of the experimental workflow. Embryos from AB/TL line were collected at 0 hour post fertilization (hpf). At 6 hpf, the exterior chorion membranes of embryos were sterilized through chemical treatment and embryos were maintained under germ-free (GF) conditions until 3 days post fertilization (dpf). At 3 dpf, when larvae had shed the chorion and developed open mouths and gastrointestinal tracts, the conventionalized (CONVD) group was generated by introducing microbes from the conventionally reared group. *M. marinum* Mma20 or *M. avium* MAC 101 bacterial systemic microinjections were performed 6 hours after group division to allow stabilization of microbial conditions prior to infection. The larvae from GF and CONVD group were systemically infected with Mma20 or MAC 101 bacteria via microinjection into the duct of Cuvier. A total of 25 successfully injected larvae for each treatment group were included for the following analysis. Survival rate monitoring was performed at 20, 40 and 48 hours post injection (hpi). **(B–D)** Kaplan Meier plots of larval survival rates after infection with increasing infectious burdens of Mma20, 250 CFU **(B)**, 500 CFU **(C)** and 1000 CFU **(D)**. **(E–G)** Kaplan Meier plots of larval survival rates after infection with increasing infectious burdens of MAC 101, 1000 CFU **(E)**, 2500 CFU **(F)** and 5000 CFU **(G)**. Data were collected from three independent experiments. Kaplan-Meier survival curve represented pooled data from all three experiments. Statistical curve comparisons by Mantel-Cox test; ns, non-significant.

To assess whether microbiome colonization alters host susceptibility to nontuberculous mycobacterial infection, we systemically injected *M. marinum* Mma20 and *M. avium* MAC 101 bacteria to the GF and CONVD larvae (**Figure 5A**). Survival rates for all groups after Mma20 and MAC 101 infection showed no significant differences between microbial conditions, indicating that early mortality was not a sensitive readout of microbiome-dependent effects in this setting in contrast to the case with *E.coli* (**Figures 5B–G**).

**Figure 6:**
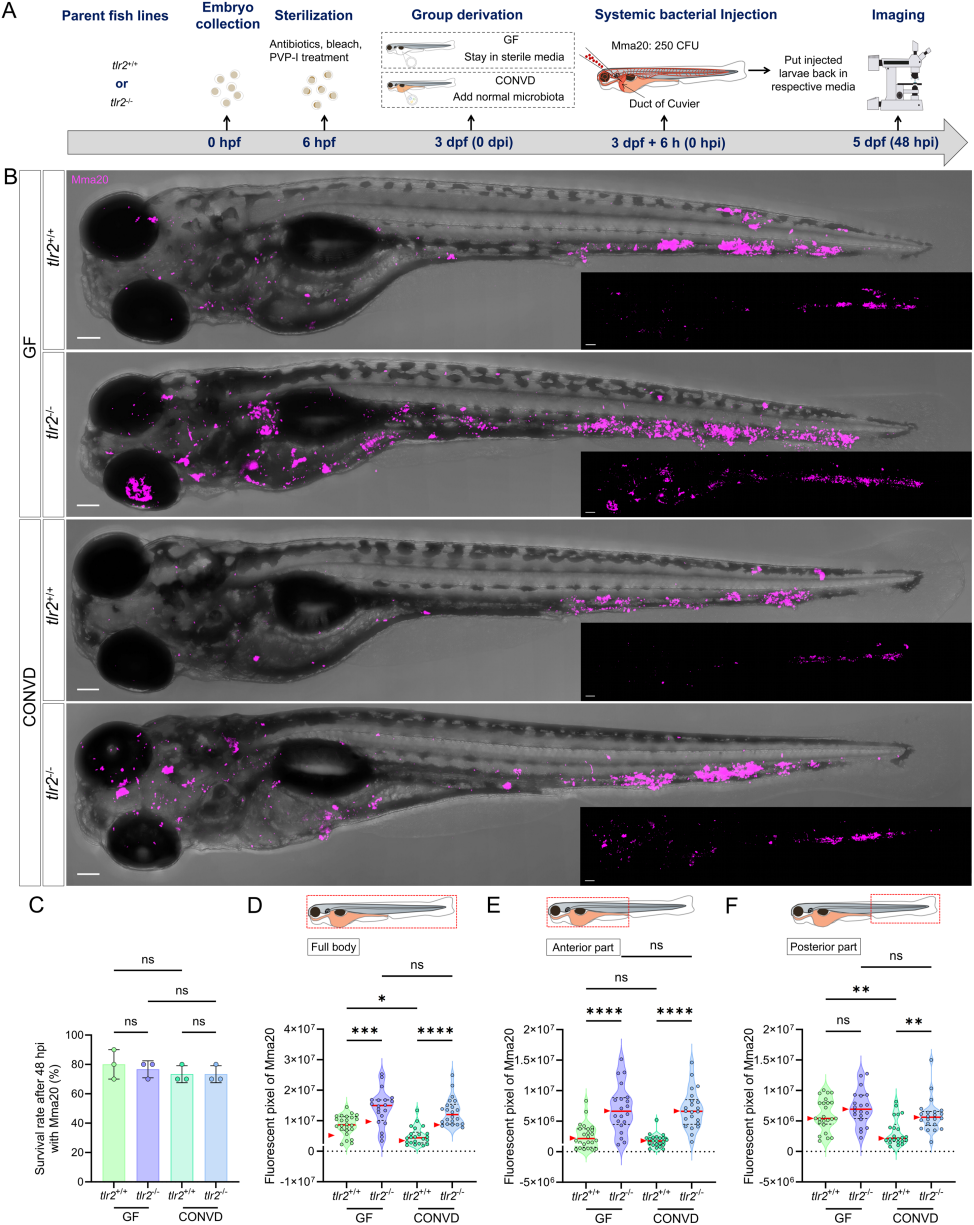
Bacterial burden of *M. marinum* Mma20 of *tlr2* mutant and wild-type zebrafish larvae after systemic infection under the germ-free or conventionalized condition. **(A)** Schematic overview of the experimental workflow. At 3 days post fertilization (dpf), *tlr2* mutant and wild-type larvae were maintained as germ-free (GF) or conventionalized (CONVD) by exposure to a defined microbial community. DsRed-labeled *M. marinum* Mma20 bacterial systemic microinjections were performed 6 hours after group division to allow stabilization of microbial conditions prior to infection and injected larvae were collected at 48 hours post injection (hpi) for confocal imaging. **(B)** Representative fluorescence images showing Mma20 dissemination to both the anterior and posterior regions of the larvae. Bacteria are shown in magenta. Scale bar: 100 µm. **(C)** Survival rates for all groups after Mma20 infection showing no significant differences between genotypes or microbial conditions. The results are based on 3 independent experiments. Error bars represent mean ± SD. **(D–F)** Quantification of Mma20 bacterial burden (fluorescent pixel count) in the full body **(D)**, anterior region **(E)** and posterior region **(F)**. The data from GF *tlr2*^+/+^ (n=24) group, GF *tlr2*^-/-^ (n=22) group, CONVD *tlr2*^+/+^ (n=20) group and CONVD *tlr2*^-/-^ (n=22) group are based on three independent experiments. Statistical significant difference was determined by one-way ANOVA, red arrows point to the median, ns, non-significant, *P < 0.05, **P < 0.01, ***P < 0.001, ****P < 0.0001.

**Figure 7:**
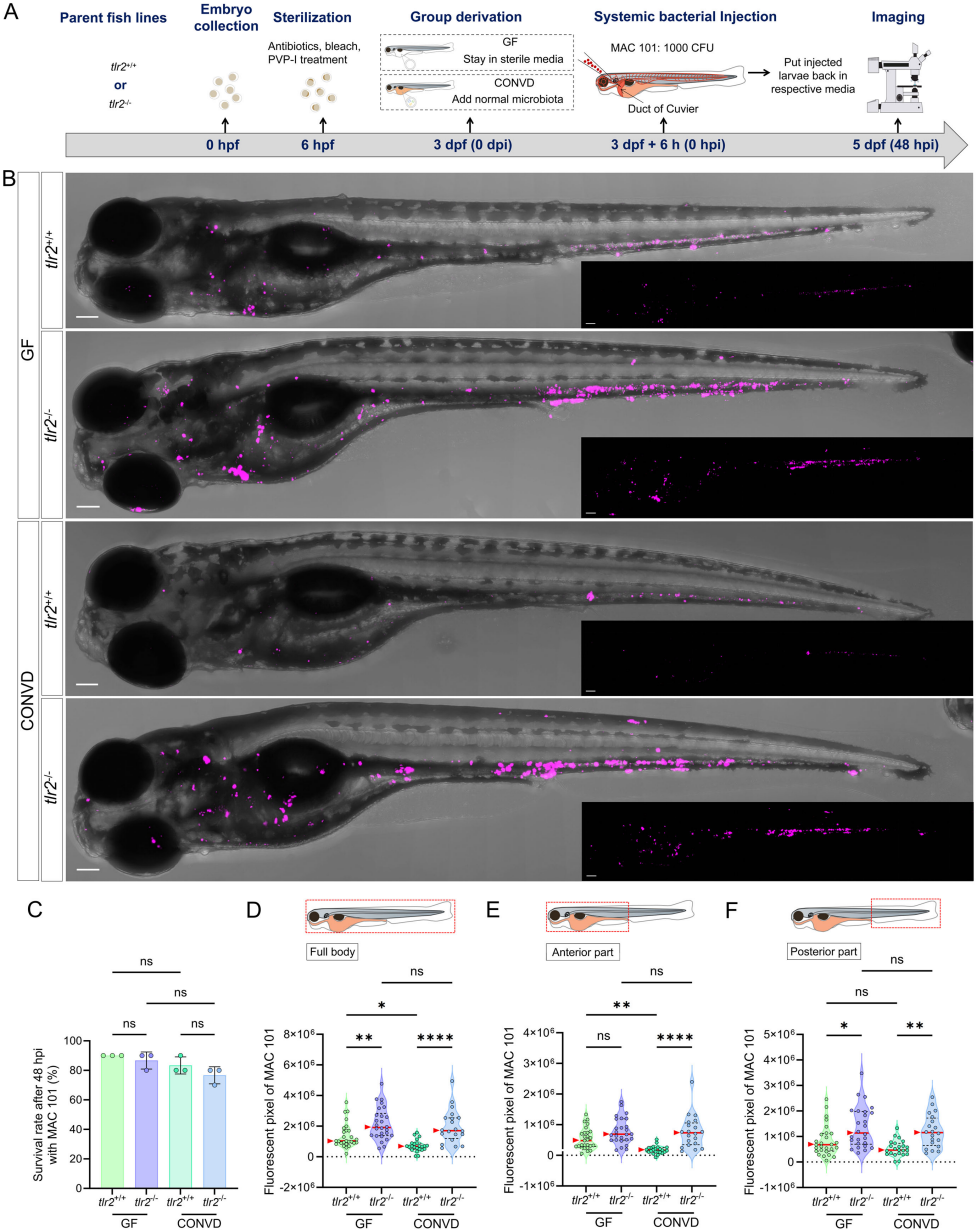
Bacterial burden of *M. avium* MAC 101 of *tlr2* mutant and wild-type zebrafish larvae after systemic infection under the germ-free or conventionalized condition. **(A)** Schematic overview of the experimental workflow. At 3 days post fertilization (dpf), *tlr2* mutant and wild-type larvae were maintained as germ-free (GF) or conventionalized (CONVD) by exposure to a defined microbial community. mCherry-labeled *M. avium* MAC 101 bacterial systemic microinjections were performed 6 hours later to allow stabilization of microbial conditions prior to infection and injected larvae were collected at 48 hours post injection (hpi) for confocal imaging. **(B)** Representative fluorescence images showing MAC 101 dissemination to both the anterior and posterior regions of the larvae. Bacteria are shown in magenta. Scale bar: 100 µm. **(C)** Survival rates for all groups after MAC 101 infection showing no significant differences between genotypes or microbial conditions. The results are based on 3 independent experiments. Error bars represent mean ± SD. **(D–F)** Quantification of MAC 101 bacterial burden (fluorescent pixel count) in the full body **(D)**, anterior region **(E)** and posterior region **(F)**. The data from GF *tlr2*^+/+^ (n=27) group, GF *tlr2*^-/-^ (n=26) group, CONVD *tlr2*^+/+^ (n=23) group and CONVD *tlr2*^-/-^ (n=20) group are based on three independent experiments. Statistical significant difference was determined by one-way ANOVA, red arrows point to the median, ns, non-significant, *P < 0.05, **P < 0.01, ****P < 0.0001.

To further determine the contribution of TLR2 and the microbiome to systemic host defense of nontuberculous mycobacteria, we injected fluorescent strains (Mma20 DsRed and MAC 101 mCherry) to the *tlr2* wild-type and mutant larvae under GF and CONVD conditions and performed confocal imaging at 2 days post infection (dpi) (**Figures 6A, B**, **7A, B**). Consistent with the initial survival experiments, no significant survival differences were detected across conditions at 2 dpi for either Mma20 or MAC 101 infections (**Figure 6C**). In contrast, quantitative imaging revealed robust genotype- and microbiome-dependent effects on bacterial proliferation. For Mma20 infection, *tlr2* mutants exhibited significantly higher bacterial burden than wild-type larvae in the whole body and anterior region under both GF and CONVD conditions (**Figures 6D, E**). In the posterior region, increased burden in *tlr2* mutants was evident specifically under CONVD conditions (**Figure 6F**). When comparing microbial states within each genotype, GF wild-type larvae carried a higher Mma20 burden than CONVD wild-type larvae, whereas this microbiome-associated reduction was not observed in *tlr2* mutants, suggesting that microbiome-mediated restriction of Mma20 growth requires functional TLR2 signaling.

A similar pattern was observed during MAC 101 infection. Although survival remained unchanged across groups (**Figure 7C**), *tlr2* mutants showed increased bacterial burden relative to wild type in the whole body and posterior region under both GF and CONVD conditions (**Figures 7D, F**). In the anterior region, the burden increase in *tlr2* mutants was apparent only under CONVD conditions (**Figure 7E**). As with Mma20, GF wild-type larvae exhibited higher MAC 101 burden than CONVD wild-type larvae, whereas no significant difference was detected in *tlr2* mutants, again indicating that microbiome-dependent control of bacterial proliferation is blunted in the absence of TLR2.

Taken together, these results demonstrate that loss of TLR2 consistently permits enhanced proliferation of both Mma20 and MAC 101, and that microbiome colonization restricts mycobacterial growth in wild-type larvae but not in *tlr2* mutants. This supports a model in which TLR2-dependent signaling contributes directly to systemic anti-mycobacterial defense, while the microbiome provides an additional layer of protection that is at least partly TLR2-dependent, potentially through priming or tuning of innate immune effector programs that limit bacterial expansion.

## 4 Discussion

### 4.1 Microbiome colonization attenuates early mortality after systemic *E. coli* infection and buffers infection-induced metabolic suppression

In this study, we demonstrate that microbiome colonization confers rapid and robust protection against early mortality following systemic *E. coli* infection in zebrafish larvae. While GF and CONVD larvae exhibited comparable survival at low infectious burden, the presence of a microbiome significantly reduced early mortality at higher bacterial doses (**Figure 1**). Our results show that microbial colonization increases the host’s tolerance to systemic bacterial challenge during early infection. The importance of a healthy microbiome in protecting against neonatal sepsis has been well established in humans; however, to the best of our knowledge, most studies have focused on late-onset sepsis (LOS) and necrotizing enterocolitis (NEC) (10, 39–41), whereas the potential role of early microbial colonization in protecting against early-onset sepsis (EOS) remains largely unexplored. This may well be a reflection of EOS being an inherently more difficult condition to study by some of the favored methods in the field, e.g. prospective cohort studies, which have provided significant contributions to the LOS field (40, 42), would be very difficult to undertake in EOS cohorts. Our zebrafish model most closely resembles an EOS paradigm by employing an EOS-associated pathogen *E. coli* (7), and addressing effects of very brief and early microbial colonization. Therefore our demonstration of a clear protective effect of colonization against sepsis should be seen as a first step towards recognizing that, as is the case for LOS, microbiome colonization may be important in protecting against EOS as well. Notably, this protective effect was observed even when microbiome exposure was limited to only six hours prior to infection, indicating that short-term microbial colonization is sufficient to enhance host resilience to septic challenge. These findings support the concept of microbial priming, whereby commensal microbes rapidly calibrate host immune responsiveness and improve tolerance to systemic infection during early life (43, 44).

The protective effects of microbial colonization against LOS and NEC are thought to be mediated through multiple mechanisms including enhancement of intestinal barrier function, direct and indirect immune modulation, metabolic effects and niche exclusion of potential pathogens (39, 45, 46). In our zebrafish microbiome model, transcriptomic profiling revealed that *E. coli* infection induces a substantial transcriptional response in both GF and CONVD larvae, including a large shared set of upregulated differentially expressed genes enriched for immune-related pathways. This conserved response likely represents a core infection-induced program that is largely independent of microbiome status and reflects fundamental host defense mechanisms activated upon bloodstream infection (47). However, the overall magnitude of transcriptional perturbation was markedly greater in GF larvae, suggesting that the absence of a microbiome leads to a less buffered and more extensive systemic response to infection. This observation is consistent with evidence that germ-free hosts display altered immune maturation and impaired immune homeostasis, often accompanied by dysregulated inflammatory tone and increased susceptibility to infection (48–50). Future studies should focus on protein-level validation and cell-type-specific analysis to provide deeper insight into the cellular basis of microbiome-mediated immune modulation.

A key finding of this work is that microbiome colonization selectively modulates the metabolic dimension of the host response to infection. While immune activation pathways were similarly enriched among upregulated genes under both GF and CONVD conditions, downregulated genes displayed pronounced microbiome-dependent differences. In particular, lipid metabolic processes and multiple carbohydrate and fatty acid metabolism pathways were strongly enriched among downregulated genes only in GF larvae (**Figures 2**, **3**). This indicates that systemic *E. coli* infection induces a broad repression of host metabolic programs in the absence of a microbiome, whereas microbial colonization buffers against infection-induced metabolic suppression. These results suggest that the microbiome helps uncouple effective immune activation from widespread metabolic shutdown, thereby preserving metabolic homeostasis during septic challenge. Mechanistically, this buffering effect may arise from continuous microbial signaling that sets immune activation thresholds, preventing excessive inflammatory spillover into metabolic regulation (51, 52). Alternatively, microbiome-derived metabolites may directly support host energy metabolism or mitochondrial function during infection, thereby reducing the metabolic cost of immune activation. Although whole-body RNA sequencing limits tissue-specific resolution, our data highlight the microbiome as a critical modulator of immunometabolic balance during systemic infection. Future studies employing tissue-resolved or single-cell approaches will be essential to delineate how microbial signals coordinate immune and metabolic responses at the cellular level.

### 4.2 Protective roles of TLR2 and the microbiome in restricting mycobacterial proliferation after systemic infection

We have previously shown that the larval innate immune system setpoint is affected by bacterial colonization by transcriptional regulation of the Toll-like receptor (TLR) adaptor molecule myd88 (28), but have not previously addressed this phenomenon under infectious conditions. Therefore, we further investigated how the microbiome and TLR2 contribute to host defense against systemic nontuberculous mycobacterial infection. Using fluorescently labeled *M. marinum* and *M. avium*, we show that both TLR2 deficiency and the absence of a microbiome result in increased bacterial burden following bloodstream infection. Although survival rates were not significantly altered within the experimental time frame, bacterial proliferation was consistently enhanced in *tlr2* mutant larvae, underscoring a critical role for TLR2-dependent signaling in restricting mycobacterial growth. Future studies could employ pharmacological approaches (e.g., TLR2 agonists or inhibitors) in both CONVD and GF larvae to further validate this conclusion.

Importantly, microbiome-dependent protection against mycobacterial proliferation was evident in wild-type larvae but was diminished or lost in the absence of TLR2. Under wild-type conditions, GF larvae displayed higher bacterial burden than CONVD larvae, whereas this difference was no longer apparent in *tlr2* mutants. These findings suggest that part of the protective effect conferred by the microbiome relies on intact TLR2 signaling. One plausible explanation is that commensal microbes provide continuous low-level pattern-recognition signals that condition innate immune cells, particularly macrophages, toward a more effective antimicrobial state (22, 53). In the absence of TLR2, this microbiome-driven immune conditioning may be compromised, resulting in reduced control of intracellular mycobacteria. It is also noteworthy that TLR2 deficiency itself has been shown to alter commensal microbial composition and diversity in both zebrafish and mice model (54, 55), which may partly explain the reduced infection control of the microbiome in *tlr2* mutants. Future studies could focus on comparing the specific microbial taxa that differ between *tlr2* wild-type and mutant larvae to identify the key bacterial taxa that confer protection against mycobacterial infection. Uncovering these key protective genera could provide new insights for microbiome-based therapeutic strategies.

Spatial analysis further revealed region-specific differences in bacterial burden, as well as pathogen-specific patterns between *M. marinum* and *M. avium*. These differences may reflect distinct tissue microenvironments, macrophage recruitment dynamics, or intrinsic differences in bacterial replication strategies. While survival was not affected under the tested conditions, an increased bacterial burden likely represents an early indicator of impaired immune control that could translate into worsened outcomes at later stages or under higher infectious pressure.

Together, our findings highlight complementary and interdependent roles for TLR2 and the microbiome in systemic host defense against mycobacterial infection. TLR2 provides a key innate sensing pathway required for effective bacterial restriction, while the microbiome enhances systemic resistance by modulating immune tone and antimicrobial capacity. These results underscore the importance of host-microbiome-immune crosstalk in shaping infection outcomes. The translational relevance of our findings is supported by a growing body of human clinical evidence. In preterm infants, gut dysbiosis characterized by reduced microbial diversity and overgrowth of pathobionts has been consistently associated with increased sepsis risk (56, 57). This parallel between our experimental model and clinical observations reinforces the notion that the interplay between host immunity and the microbiome is a critical determinant of infection susceptibility in early life. Accordingly, targeting microbiome-derived signals or TLR2-associated pathways may offer novel strategies to improve host resistance to bloodstream infections caused by both opportunistic and pathogenic bacteria.

## 5 Conclusion

Using a gnotobiotic zebrafish larval model, we show that early microbiome colonization provides rapid protection against early mortality following systemic *E. coli* infection. RNA-seq revealed a conserved core immune activation program in both GF and CONVD larvae, but the absence of a microbiome was associated with a broader transcriptional response and stronger repression of metabolic pathways, suggesting that commensal microbes buffer infection-induced metabolic suppression. Extending this framework to nontuberculous mycobacteria, imaging-based quantification demonstrated increased bacterial burden in GF larvae and in *tlr2* mutants, and microbiome-mediated restriction of mycobacterial proliferation was evident in wild-type but not TLR2-deficient hosts. Together, these findings identify microbiome colonization as a key determinant of systemic infection outcomes in early life and highlight TLR2 as an important node linking microbial cues to effective control of mycobacterial growth. Future work should focus on identifying the specific microbial cues driving TLR2-dependent protection and to assess its translational relevance in human diseases.

## Data Availability

The raw sequencing data and analyzed expression data of RNAseq are available in the NCBI Gene Expression Omnibus (GEO) database under accession number GSE325739.
