# Supplementary material for: The microbiome protects against septic hyperinflammation and bacterial proliferation in a zebrafish model of blood infection with Escherichia coli and mycobacteria
Source: Front Immunol. 2026 Jul 2;17:1837804. doi: 10.3389/fimmu.2026.1837804 (PMC13372636; doi:10.3389/fimmu.2026.1837804)
Supplement: Supplementary file 1 [file DataSheet1.docx]

**Supplementary figure 1. The colony forming units (CFU) in each individual embryo after systemic *E. coli* infection**

At 2, 4, and 18 hours post injection (hpi), CFU counts were determined for individual larvae (n=8 per group per time point). Data are shown as mean ± SEM. No significant differences were observed at 2 hpi or 18 hpi, whereas a trend toward higher bacterial burden in GF larvae was observed at 4 hpi. This time point was therefore selected for transcriptomic analysis. GF, germ-free group; CONVD, conventionalized group; hpi, hours post injection; CFU, colony forming units.

**
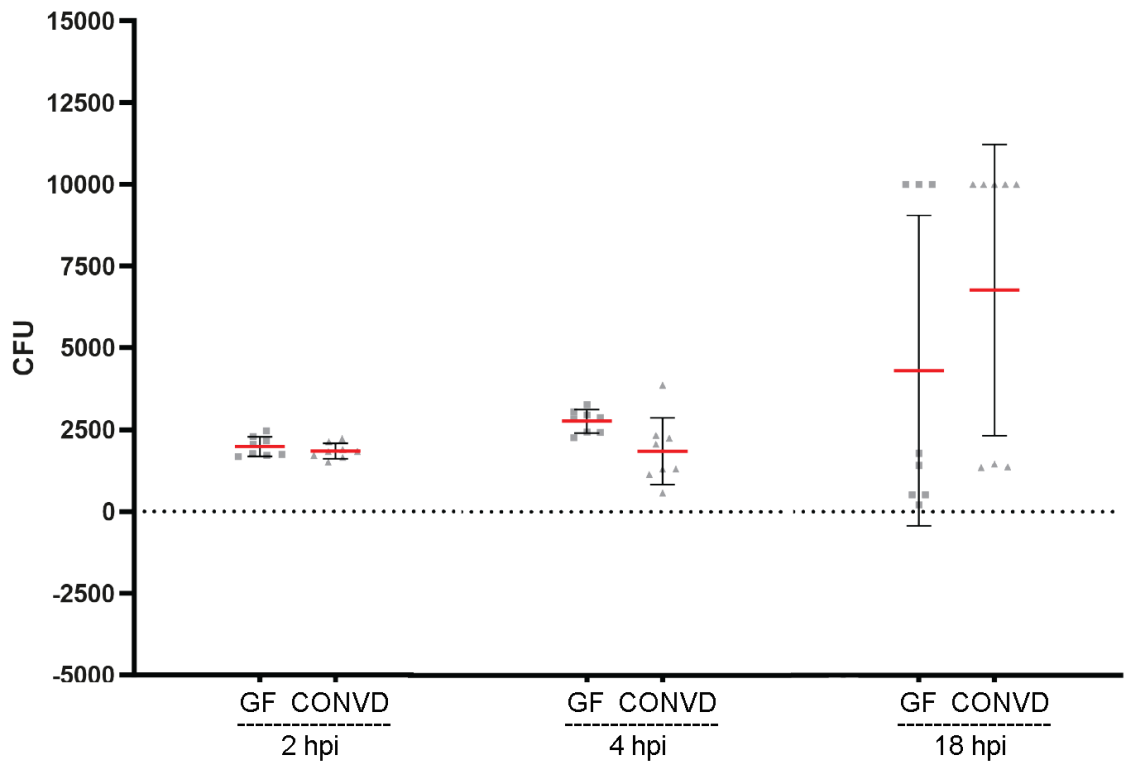
**

**Supplementary table 1. Differentially expressed genes (DEGs) in the representative pathway in systemic *E. coli* infected zebrafish larvae compared to the PBS injected controls under the GF condition**

| Pathway | Ensembl ID | Gene name | log2 Fold Change | FDR p-value |
| --- | --- | --- | --- | --- |
| Toll like receptor signaling | [ENSDARG00000044415](https://www.ensembl.org/id/ENSDARG00000044415) | *tlr5a* | 1.545009199 | 1.06907E-05 |
|  | [ENSDARG00000052322](https://www.ensembl.org/id/ENSDARG00000052322) | *tlr5b* | 1.848595983 | 8.20728E-45 |
|  | [ENSDARG00000010169](https://www.ensembl.org/id/ENSDARG00000010169) | *myd88* | 1.001108328 | 4.36782E-07 |
|  | [ENSDARG00000102762](https://www.ensembl.org/id/ENSDARG00000102762) | *pik3r5* | 1.710517784 | 3.05991E-07 |
|  | [ENSDARG00000103021](https://www.ensembl.org/id/ENSDARG00000103021) | *fadd* | 0.687502559 | 0.003254416 |
|  | [ENSDARG00000058325](https://www.ensembl.org/id/ENSDARG00000058325) | *casp8* | 1.076933698 | 2.84217E-08 |
|  | [ENSDARG00000053131](https://www.ensembl.org/id/ENSDARG00000053131) | *irak3* | 6.749013058 | 8.5813E-118 |
|  | [ENSDARG00000022000](https://www.ensembl.org/id/ENSDARG00000022000) | *traf3* | 2.450080497 | 2.7401E-110 |
|  | [ENSDARG00000070606](https://www.ensembl.org/id/ENSDARG00000070606) | *ikbke* | 0.779510798 | 2.297E-08 |
|  | [ENSDARG00000076251](https://www.ensembl.org/id/ENSDARG00000076251) | *irf3* | 2.116991595 | 5.6729E-08 |
|  | [ENSDARG00000045661](https://www.ensembl.org/id/ENSDARG00000045661) | *irf7* | 3.429934573 | 2.67805E-74 |
|  | [ENSDARG00000043531](https://www.ensembl.org/id/ENSDARG00000043531) | *jun* | 1.29299004 | 2.51175E-71 |
|  | [ENSDARG00000067850](https://www.ensembl.org/id/ENSDARG00000067850) | *jund* | 1.001413165 | 2.17446E-25 |
|  | [ENSDARG00000074378](https://www.ensembl.org/id/ENSDARG00000074378) | *junba* | 3.014769972 | 9.8318E-138 |
|  | [ENSDARG00000104773](https://www.ensembl.org/id/ENSDARG00000104773) | *junbb* | 3.004885489 | 1.575E-129 |
|  | [ENSDARG00000007823](https://www.ensembl.org/id/ENSDARG00000007823) | *atf3* | 4.173152669 | 2.2058E-245 |
|  | [ENSDARG00000042577](https://www.ensembl.org/id/ENSDARG00000042577) | *batf3* | 3.637578579 | 0.000193563 |
|  | [ENSDARG00000040623](https://www.ensembl.org/id/ENSDARG00000040623) | *fosl2* | 2.086215592 | 4.82381E-71 |
|  | [ENSDARG00000031683](https://www.ensembl.org/id/ENSDARG00000031683) | *fosab* | 3.512134826 | 9.9257E-106 |
|  | [ENSDARG00000015355](https://www.ensembl.org/id/ENSDARG00000015355) | *fosl1a* | 4.793606483 | 1.7807E-108 |
|  | [ENSDARG00000105261](https://www.ensembl.org/id/ENSDARG00000105261) | *nfkb1* | 1.367260037 | 1.3767E-22 |
|  | [ENSDARG00000038687](https://www.ensembl.org/id/ENSDARG00000038687) | *nfkb2* | 2.695596426 | 9.6927E-266 |
|  | [ENSDARG00000005481](https://www.ensembl.org/id/ENSDARG00000005481) | *nfkbiaa* | 4.196678526 | 4.3116E-282 |
|  | [ENSDARG00000007693](https://www.ensembl.org/id/ENSDARG00000007693) | *nfkbiab* | 2.832700179 | 6.8093E-153 |
|  | [ENSDARG00000000857](https://www.ensembl.org/id/ENSDARG00000000857) | *mapk14a* | 0.690899246 | 4.99596E-09 |
|  | [ENSDARG00000054968](https://www.ensembl.org/id/ENSDARG00000054968) | *cd40* | 2.854926279 | 1.26887E-37 |
|  | [ENSDARG00000025949](https://www.ensembl.org/id/ENSDARG00000025949) | *irak1* | 0.706400914 | 0.004699851 |
|  | [ENSDARG00000098696](https://www.ensembl.org/id/ENSDARG00000098696) | *rela* | 0.615387264 | 0.000209324 |
| Apoptosis | [ENSDARG00000068367](https://www.ensembl.org/id/ENSDARG00000068367) | *nfkbie* | 2.880760277 | 2.0185E-128 |
|  | [ENSDARG00000007693](https://www.ensembl.org/id/ENSDARG00000007693) | *nfkbiab* | 2.832700179 | 6.8093E-153 |
|  | [ENSDARG00000105261](https://www.ensembl.org/id/ENSDARG00000105261) | *nfkb1* | 1.367260037 | 1.3767E-22 |
|  | [ENSDARG00000018569](https://www.ensembl.org/id/ENSDARG00000018569) | *tnfrsf1a* | 1.137108374 | 9.58524E-40 |
|  | [ENSDARG00000089307](https://www.ensembl.org/id/ENSDARG00000089307) | *pmaip1* | 2.563441817 | 8.59623E-47 |
|  | [ENSDARG00000058325](https://www.ensembl.org/id/ENSDARG00000058325) | *casp8* | 1.076933698 | 2.84217E-08 |
|  | [ENSDARG00000004325](https://www.ensembl.org/id/ENSDARG00000004325) | *casp9* | 0.669737816 | 8.33302E-06 |
|  | [ENSDARG00000044125](https://www.ensembl.org/id/ENSDARG00000044125) | *txn* | 0.611697988 | 1.13629E-06 |
|  | [ENSDARG00000043586](https://www.ensembl.org/id/ENSDARG00000043586) | *fas* | 1.998604398 | 1.56869E-07 |
|  | [ENSDARG00000013598](https://www.ensembl.org/id/ENSDARG00000013598) | *tnfb* | 6.458530571 | 4.79822E-59 |
|  | [ENSDARG00000018569](https://www.ensembl.org/id/ENSDARG00000018569) | *tnfrsf1a* | 1.137108374 | 9.58524E-40 |
|  | [ENSDARG00000070165](https://www.ensembl.org/id/ENSDARG00000070165) | *tnfrsf1b* | 0.623271678 | 0.001985681 |
|  | [ENSDARG00000069482](https://www.ensembl.org/id/ENSDARG00000069482) | *traf1* | 2.351034407 | 1.0122E-06 |
|  | [ENSDARG00000022000](https://www.ensembl.org/id/ENSDARG00000022000) | *traf3* | 2.450080497 | 2.7401E-110 |
|  | [ENSDARG00000103021](https://www.ensembl.org/id/ENSDARG00000103021) | *fadd* | 0.687502559 | 0.003254416 |
|  | [ENSDARG00000055966](https://www.ensembl.org/id/ENSDARG00000055966) | *cflara* | 1.564244285 | 4.69026E-33 |
|  | [ENSDARG00000030087](https://www.ensembl.org/id/ENSDARG00000030087) | *nfkbib* | 1.898020492 | 4.5818E-159 |
|  | [ENSDARG00000104172](https://www.ensembl.org/id/ENSDARG00000104172) | *diabloa* | -1.29610401 | 5.45796E-13 |
|  | [ENSDARG00000070165](https://www.ensembl.org/id/ENSDARG00000070165) | *tnfrsf1b* | 0.623271678 | 0.001985681 |
|  | [ENSDARG00000098696](https://www.ensembl.org/id/ENSDARG00000098696) | *rela* | 0.615387264 | 0.000209324 |
|  | [ENSDARG00000087993](https://www.ensembl.org/id/ENSDARG00000087993) | *bada* | 0.829218115 | 0.028988677 |
|  | [ENSDARG00000044356](https://www.ensembl.org/id/ENSDARG00000044356) | *tp63* | -0.648684698 | 1.8338E-13 |
| Metabolic pathways | [ENSDARG00000099960](https://www.ensembl.org/id/ENSDARG00000099960) | *elovl1a* | 1.143614295 | 7.19292E-19 |
|  | [ENSDARG00000002634](https://www.ensembl.org/id/ENSDARG00000002634) | *b4galt1* | 0.920288321 | 4.5222E-10 |
|  | [ENSDARG00000068515](https://www.ensembl.org/id/ENSDARG00000068515) | *chs1* | 1.147306783 | 2.6792E-08 |
|  | [ENSDARG00000037873](https://www.ensembl.org/id/ENSDARG00000037873) | *cyp3c3_1* | 1.848614069 | 7.98708E-05 |
|  | [ENSDARG00000033999](https://www.ensembl.org/id/ENSDARG00000033999) | *cyp26a1* | 0.840748735 | 1.90396E-10 |
|  | [ENSDARG00000056029](https://www.ensembl.org/id/ENSDARG00000056029) | *cyp26c1* | 0.624970461 | 7.66938E-06 |
|  | [ENSDARG00000092660](https://www.ensembl.org/id/ENSDARG00000092660) | *cyp27c1* | 0.906622072 | 4.19071E-09 |
|  | [ENSDARG00000044002](https://www.ensembl.org/id/ENSDARG00000044002) | *cyp2x7* | 0.738265351 | 5.37279E-06 |
|  | [ENSDARG00000013721](https://www.ensembl.org/id/ENSDARG00000013721) | *g6pca.2* | -0.597861895 | 0.000222731 |
|  | [ENSDARG00000010752](https://www.ensembl.org/id/ENSDARG00000010752) | *acsl4b* | 0.62493979 | 5.18344E-10 |
|  | [ENSDARG00000093193](https://www.ensembl.org/id/ENSDARG00000093193) | *chia.6_2* | 0.950744734 | 0.004164843 |
|  | [ENSDARG00000055652](https://www.ensembl.org/id/ENSDARG00000055652) | *aclyb* | -0.555368449 | 0.042079448 |
|  | [ENSDARG00000075931](https://www.ensembl.org/id/ENSDARG00000075931) | *acsl5* | -0.577714337 | 0.00321237 |
|  | [ENSDARG00000014138](https://www.ensembl.org/id/ENSDARG00000014138) | *acot8* | -0.699189365 | 0.037224358 |
|  | [ENSDARG00000053774](https://www.ensembl.org/id/ENSDARG00000053774) | *alpi.2* | -0.89345003 | 0.040084154 |
|  | [ENSDARG00000042293](https://www.ensembl.org/id/ENSDARG00000042293) | *ca4b* | -1.097090575 | 0.002305396 |
|  | [ENSDARG00000069018](https://www.ensembl.org/id/ENSDARG00000069018) | *cyp7a1* | -0.633228478 | 0.002925466 |
|  | [ENSDARG00000035506](https://www.ensembl.org/id/ENSDARG00000035506) | *entpd2a.1* | -0.823609959 | 0.001856831 |
|  | [ENSDARG00000071429](https://www.ensembl.org/id/ENSDARG00000071429) | *tdo2a* | -0.812554729 | 5.74679E-05 |
|  | [ENSDARG00000023176](https://www.ensembl.org/id/ENSDARG00000023176) | *tdo2b* | -1.850108714 | 0.003463091 |
| IFN pathway | [ENSDARG00000040332](https://www.ensembl.org/id/ENSDARG00000040332) | *crfb2* | 0.671752302 | 0.044088656 |
|  | [ENSDARG00000006266](https://www.ensembl.org/id/ENSDARG00000006266) | *stat1a* | 0.968598519 | 7.47768E-18 |
|  | [ENSDARG00000022712](https://www.ensembl.org/id/ENSDARG00000022712) | *stat3* | 0.871161739 | 3.30728E-26 |
|  | [ENSDARG00000028731](https://www.ensembl.org/id/ENSDARG00000028731) | *stat4* | 1.40415886 | 2.86281E-27 |
|  | [ENSDARG00000040465](https://www.ensembl.org/id/ENSDARG00000040465) | *irf2* | 1.16878394 | 1.32179E-15 |
|  | [ENSDARG00000076251](https://www.ensembl.org/id/ENSDARG00000076251) | *irf3* | 2.116991595 | 5.6729E-08 |
|  | [ENSDARG00000045661](https://www.ensembl.org/id/ENSDARG00000045661) | *irf7* | 3.429934573 | 2.67805E-74 |
|  | [ENSDARG00000056407](https://www.ensembl.org/id/ENSDARG00000056407) | *irf8* | 2.91585594 | 6.55059E-39 |
|  | [ENSDARG00000016457](https://www.ensembl.org/id/ENSDARG00000016457) | *irf9* | 1.754093609 | 6.55427E-41 |
|  | [ENSDARG00000027658](https://www.ensembl.org/id/ENSDARG00000027658) | *irf10* | 2.45508554 | 2.41989E-09 |
|  | [ENSDARG00000100515](https://www.ensembl.org/id/ENSDARG00000100515) | *usp18* | 1.356808038 | 3.71939E-33 |
|  | [ENSDARG00000024789](https://www.ensembl.org/id/ENSDARG00000024789) | *mxc* | 4.686314093 | 6.72E-222 |
|  | [ENSDARG00000014427](https://www.ensembl.org/id/ENSDARG00000014427) | *mxe* | 3.487068935 | 9.90318E-10 |
|  | [ENSDARG00000026049](https://www.ensembl.org/id/ENSDARG00000026049) | *mxf* | 7.911131115 | 5.9425E-21 |
|  | [ENSDARG00000052396](https://www.ensembl.org/id/ENSDARG00000052396) | *pkz* | 3.440095787 | 1.99392E-14 |
|  | [ENSDARG00000075643](https://www.ensembl.org/id/ENSDARG00000075643) | *ifi35* | 1.563550641 | 1.8232E-10 |
|  | [ENSDARG00000033227](https://www.ensembl.org/id/ENSDARG00000033227) | *lect2l* | 2.539225385 | 6.07193E-59 |
|  | [ENSDARG00000053136](https://www.ensembl.org/id/ENSDARG00000053136) | *b2m* | 2.277812518 | 6.85911E-19 |
|  | [ENSDARG00000033144](https://www.ensembl.org/id/ENSDARG00000033144) | *psme2* | 1.413848531 | 6.60375E-10 |
|  | [ENSDARG00000075963](https://www.ensembl.org/id/ENSDARG00000075963) | *mhc1uba* | 1.397958741 | 0.001668833 |
|  | [ENSDARG00000055278](https://www.ensembl.org/id/ENSDARG00000055278) | *cfb* | 0.972224152 | 8.18641E-13 |
|  | [ENSDARG00000057121](https://www.ensembl.org/id/ENSDARG00000057121) | *c7b* | 4.052995692 | 1.0642E-162 |
|  | [ENSDARG00000012694](https://www.ensembl.org/id/ENSDARG00000012694) | *c3a.1* | 1.856833498 | 2.77364E-35 |
|  | [ENSDARG00000043719](https://www.ensembl.org/id/ENSDARG00000043719) | *c3a.6* | 1.710418611 | 1.43564E-27 |
|  | [ENSDARG00000040332](https://www.ensembl.org/id/ENSDARG00000040332) | *crfb2* | 0.671752302 | 0.044088656 |
|  | [ENSDARG00000075643](https://www.ensembl.org/id/ENSDARG00000075643) | *ifi44a7* | 1.563550641 | 1.8232E-10 |
|  | [ENSDARG00000093303](https://www.ensembl.org/id/ENSDARG00000093303) | *ifi44a8* | 1.974427417 | 7.15601E-26 |
|  | [ENSDARG00000043899](https://www.ensembl.org/id/ENSDARG00000043899) | *psma6l* | 0.835829547 | 0.024872217 |
|  | [ENSDARG00000052207](https://www.ensembl.org/id/ENSDARG00000052207) | *c3a.3* | 0.839820201 | 9.7624E-05 |
|  | [ENSDARG00000093198](https://www.ensembl.org/id/ENSDARG00000093198) | *c3a.4* | 2.685708371 | 0.00068618 |
|  | [ENSDARG00000100599](https://www.ensembl.org/id/ENSDARG00000100599) | *c1s* | 2.051157534 | 0.008732851 |
|  | [ENSDARG00000006560](https://www.ensembl.org/id/ENSDARG00000006560) | *irf4a* | -2.099356073 | 0.04904924 |
|  | [ENSDARG00000033382](https://www.ensembl.org/id/ENSDARG00000033382) | *ifi44f2* | -0.462932335 | 0.009703108 |
|  | [ENSDARG00000025903](https://www.ensembl.org/id/ENSDARG00000025903) | *lgals9l1* | -1.220484736 | 4.72812E-07 |
| Cytokine-cytokine receptor interaction | [ENSDARG00000058389](https://www.ensembl.org/id/ENSDARG00000058389) | *ccl19a.1* | 2.556672559 | 7.79384E-17 |
|  | [ENSDARG00000039351](https://www.ensembl.org/id/ENSDARG00000039351) | *ccl19b* | 4.454152591 | 1.15614E-60 |
|  | [ENSDARG00000094511](https://www.ensembl.org/id/ENSDARG00000094511) | *ccl20b* | 2.178610749 | 0.022697222 |
|  | [ENSDARG00000101040](https://www.ensembl.org/id/ENSDARG00000101040) | *ccl20a.3* | 7.364631603 | 8.34654E-39 |
|  | [ENSDARG00000090873](https://www.ensembl.org/id/ENSDARG00000090873) | *ccl34a.4* | 3.748738373 | 1.3274E-34 |
|  | [ENSDARG00000103466](https://www.ensembl.org/id/ENSDARG00000103466) | *ccl35.1* | 5.49778464 | 3.23632E-12 |
|  | [ENSDARG00000041917](https://www.ensembl.org/id/ENSDARG00000041917) | *ccl38a.4* | 7.284601668 | 0.02771148 |
|  | [ENSDARG00000104795](https://www.ensembl.org/id/ENSDARG00000104795) | *cxcl8a* | 4.933866107 | 9.5874E-187 |
|  | [ENSDARG00000102299](https://www.ensembl.org/id/ENSDARG00000102299) | *cxcl8b.1* | 1.45532894 | 0.004612333 |
|  | [ENSDARG00000100662](https://www.ensembl.org/id/ENSDARG00000100662) | *cxcl11.1* | 7.381268869 | 0.024550315 |
|  | [ENSDARG00000075045](https://www.ensembl.org/id/ENSDARG00000075045) | *cxcl18b* | 7.888858046 | 0.000426023 |
|  | [ENSDARG00000075163](https://www.ensembl.org/id/ENSDARG00000075163) | *cxcl20* | 1.60561436 | 2.71017E-11 |
|  | [ENSDARG00000070669](https://www.ensembl.org/id/ENSDARG00000070669) | *cxcr3.3* | 2.895604987 | 7.0514E-114 |
|  | [ENSDARG00000022000](https://www.ensembl.org/id/ENSDARG00000022000) | *traf3* | 2.450080497 | 2.7401E-110 |
|  | [ENSDARG00000077207](https://www.ensembl.org/id/ENSDARG00000077207) | *lzts2b* | 0.789865316 | 0.005178829 |
|  | [ENSDARG00000098700](https://www.ensembl.org/id/ENSDARG00000098700) | *il1b* | 7.767926806 | 1.32612E-87 |
|  | [ENSDARG00000104693](https://www.ensembl.org/id/ENSDARG00000104693) | *il6st* | 1.073439398 | 5.60699E-23 |
|  | [ENSDARG00000054542](https://www.ensembl.org/id/ENSDARG00000054542) | *il12bb* | 2.801135414 | 0.012328787 |
|  | [ENSDARG00000057180](https://www.ensembl.org/id/ENSDARG00000057180) | *il17c* | 7.657516422 | 0.0173599 |
|  | [ENSDARG00000089383](https://www.ensembl.org/id/ENSDARG00000089383) | *il1fma* | 2.811768587 | 1.76067E-05 |
|  | [ENSDARG00000102583](https://www.ensembl.org/id/ENSDARG00000102583) | *il4r.1* | 1.780034701 | 4.22616E-34 |
|  | [ENSDARG00000116052](https://www.ensembl.org/id/ENSDARG00000116052) | *il4r.2* | 0.984059225 | 0.000537751 |
|  | [ENSDARG00000074850](https://www.ensembl.org/id/ENSDARG00000074850) | *il12rb2l* | 1.639548161 | 3.88916E-21 |
|  | [ENSDARG00000058244](https://www.ensembl.org/id/ENSDARG00000058244) | *il17ra1a* | 1.119596207 | 4.57141E-15 |
|  | [ENSDARG00000009511](https://www.ensembl.org/id/ENSDARG00000009511) | *tnfa* | 8.489885842 | 0.005531637 |
|  | [ENSDARG00000013598](https://www.ensembl.org/id/ENSDARG00000013598) | *tnfb* | 6.458530571 | 4.79822E-59 |
|  | [ENSDARG00000028731](https://www.ensembl.org/id/ENSDARG00000028731) | *stat4* | 1.40415886 | 2.86281E-27 |
|  | [ENSDARG00000102211](https://www.ensembl.org/id/ENSDARG00000102211) | *csf3a* | 3.817326111 | 2.18863E-12 |
|  | [ENSDARG00000098752](https://www.ensembl.org/id/ENSDARG00000098752) | *csf3b* | 9.931271327 | 0.000540294 |
|  | [ENSDARG00000039863](https://www.ensembl.org/id/ENSDARG00000039863) | *lifrb* | 1.20325555 | 1.76609E-15 |
|  | [ENSDARG00000053624](https://www.ensembl.org/id/ENSDARG00000053624) | *csf1rb* | -2.643535936 | 3.47827E-06 |
|  | [ENSDARG00000038541](https://www.ensembl.org/id/ENSDARG00000038541) | *ccr12a* | -3.196871911 | 0.00081134 |
|  | [ENSDARG00000057633](https://www.ensembl.org/id/ENSDARG00000057633) | *cxcr4a* | -0.829298842 | 0.00529321 |
|  | [ENSDARG00000078147](https://www.ensembl.org/id/ENSDARG00000078147) | *il10* | 4.254104207 | 0.01972107 |
|  | [ENSDARG00000100383](https://www.ensembl.org/id/ENSDARG00000100383) | *il10ra* | 1.870990166 | 0.005143101 |
|  | [ENSDARG00000057323](https://www.ensembl.org/id/ENSDARG00000057323) | *e2f8* | 0.627592822 | 9.40876E-06 |
|  | [ENSDARG00000045548](https://www.ensembl.org/id/ENSDARG00000045548) | *lepb* | 4.560389746 | 0.009127986 |
|  | [ENSDARG00000041917](https://www.ensembl.org/id/ENSDARG00000041917) | *ccl38a.4* | 7.284601668 | 0.02771148 |
|  | [ENSDARG00000052361](https://www.ensembl.org/id/ENSDARG00000052361) | *il15* | -0.826275004 | 0.001362666 |
|  | [ENSDARG00000090720](https://www.ensembl.org/id/ENSDARG00000090720) | *grem1a_1* | -2.500999615 | 0.00442698 |
|  | [ENSDARG00000041959](https://www.ensembl.org/id/ENSDARG00000041959) | *cxcr4b* | -0.810968721 | 0.000351339 |
| Phagosome | [ENSDARG00000018283](https://www.ensembl.org/id/ENSDARG00000018283) | *cyba* | 0.67771808 | 1.71815E-05 |
|  | [ENSDARG00000056615](https://www.ensembl.org/id/ENSDARG00000056615) | *cybb* | 1.128572 | 1.34126E-08 |
|  | [ENSDARG00000006353](https://www.ensembl.org/id/ENSDARG00000006353) | *itga5* | 0.797199272 | 4.21465E-10 |
|  | [ENSDARG00000033735](https://www.ensembl.org/id/ENSDARG00000033735) | *ncf1* | 4.022357866 | 2.4432E-125 |
|  | [ENSDARG00000060410](https://www.ensembl.org/id/ENSDARG00000060410) | *thbs2a* | 0.592372662 | 0.007764054 |
|  | [ENSDARG00000079766](https://www.ensembl.org/id/ENSDARG00000079766) | *tap1* | 2.951289835 | 2.70392E-30 |
|  | [ENSDARG00000036787](https://www.ensembl.org/id/ENSDARG00000036787) | *tap2a* | 3.864415534 | 2.0712E-08 |
|  | [ENSDARG00000033446](https://www.ensembl.org/id/ENSDARG00000033446) | *tap2t* | 1.165757332 | 7.37834E-10 |
|  | [ENSDARG00000016939](https://www.ensembl.org/id/ENSDARG00000016939) | *itgb2* | -1.149808634 | 1.73183E-07 |
|  | [ENSDARG00000075963](https://www.ensembl.org/id/ENSDARG00000075963) | *mhc1uba* | 1.397958741 | 0.001668833 |
|  | [ENSDARG00000074656](https://www.ensembl.org/id/ENSDARG00000074656) | *ctss2.1* | -1.361634513 | 6.74085E-05 |
|  | [ENSDARG00000054202](https://www.ensembl.org/id/ENSDARG00000054202) | *hbl4* | -1.43729985 | 0.031861271 |
| Autophagy regulators | [ENSDARG00000045561](https://www.ensembl.org/id/ENSDARG00000045561) | *dram1* | 2.822024807 | 3.73028E-13 |
|  | [ENSDARG00000002663](https://www.ensembl.org/id/ENSDARG00000002663) | *optn* | 1.333631641 | 8.0337E-58 |
|  | [ENSDARG00000089399](https://www.ensembl.org/id/ENSDARG00000089399) | *tmem176l.2* | 1.444527997 | 6.96555E-17 |
| Matrix remodeling | [ENSDARG00000042816](https://www.ensembl.org/id/ENSDARG00000042816) | *mmp9* | 4.741359142 | 5.5061E-167 |
|  | [ENSDARG00000114451](https://www.ensembl.org/id/ENSDARG00000114451) | *mmp13a_2* | 3.110140986 | 4.81582E-57 |
|  | [ENSDARG00000045887](https://www.ensembl.org/id/ENSDARG00000045887) | *mmp30* | 0.649984731 | 1.94004E-07 |
|  | [ENSDARG00000001452](https://www.ensembl.org/id/ENSDARG00000001452) | *adam8a* | 0.631933119 | 0.003056645 |
|  | [ENSDARG00000057644](https://www.ensembl.org/id/ENSDARG00000057644) | *adam8b* | 1.286468436 | 2.7185E-09 |
|  | [ENSDARG00000103519](https://www.ensembl.org/id/ENSDARG00000103519) | *adamts1* | 1.039766947 | 4.40507E-13 |
|  | [ENSDARG00000021859](https://www.ensembl.org/id/ENSDARG00000021859) | *erap1b* | 0.970737921 | 0.000808653 |
|  | [ENSDARG00000010146](https://www.ensembl.org/id/ENSDARG00000010146) | *cpa2* | 2.725574624 | 1.97453E-27 |
|  | [ENSDARG00000043722](https://www.ensembl.org/id/ENSDARG00000043722) | *cpa4* | 1.303291329 | 3.07734E-13 |
|  | [ENSDARG00000075261](https://www.ensembl.org/id/ENSDARG00000075261) | *timp2b* | 2.008499631 | 3.62706E-69 |
|  | [ENSDARG00000010423](https://www.ensembl.org/id/ENSDARG00000010423) | *npsn* | -1.221031822 | 5.50534E-19 |
|  | [ENSDARG00000103878](https://www.ensembl.org/id/ENSDARG00000103878) | *anpepla* | 0.475468402 | 0.000506491 |
|  | [ENSDARG00000016718](https://www.ensembl.org/id/ENSDARG00000016718) | *mmp11b* | 0.418935972 | 0.01074472 |
|  | [ENSDARG00000008388](https://www.ensembl.org/id/ENSDARG00000008388) | *mmp14b* | 0.393342624 | 1.47398E-06 |
|  | [ENSDARG00000007709](https://www.ensembl.org/id/ENSDARG00000007709) | *adamts8a* | 1.040211696 | 6.62408E-06 |
|  | [ENSDARG00000029124](https://www.ensembl.org/id/ENSDARG00000029124) | *adamts15a* | 0.604215621 | 0.006276079 |

**Supplementary table 2. Differentially expressed genes (DEGs) in the representative pathway in systemic *E. coli* infected zebrafish larvae compared to the PBS injected controls under the CONVD condition**

| Pathway | Ensembl ID | Gene name | log2 Fold Change | FDR p-value |
| --- | --- | --- | --- | --- |
| Toll like receptor signaling | [ENSDARG00000044415](https://www.ensembl.org/id/ENSDARG00000044415) | *tlr5a* | 1.545009199 | 1.06907E-05 |
|  | [ENSDARG00000052322](https://www.ensembl.org/id/ENSDARG00000052322) | *tlr5b* | 1.848595983 | 8.20728E-45 |
|  | [ENSDARG00000010169](https://www.ensembl.org/id/ENSDARG00000010169) | *myd88* | 1.001108328 | 4.36782E-07 |
|  | [ENSDARG00000102762](https://www.ensembl.org/id/ENSDARG00000102762) | *pik3r5* | 1.710517784 | 3.05991E-07 |
|  | [ENSDARG00000103021](https://www.ensembl.org/id/ENSDARG00000103021) | *fadd* | 0.687502559 | 0.003254416 |
|  | [ENSDARG00000058325](https://www.ensembl.org/id/ENSDARG00000058325) | *casp8* | 1.076933698 | 2.84217E-08 |
|  | [ENSDARG00000053131](https://www.ensembl.org/id/ENSDARG00000053131) | *irak3* | 6.749013058 | 8.5813E-118 |
|  | [ENSDARG00000022000](https://www.ensembl.org/id/ENSDARG00000022000) | *traf3* | 2.450080497 | 2.7401E-110 |
|  | [ENSDARG00000070606](https://www.ensembl.org/id/ENSDARG00000070606) | *ikbke* | 0.779510798 | 2.297E-08 |
|  | [ENSDARG00000076251](https://www.ensembl.org/id/ENSDARG00000076251) | *irf3* | 2.116991595 | 5.6729E-08 |
|  | [ENSDARG00000045661](https://www.ensembl.org/id/ENSDARG00000045661) | *irf7* | 3.429934573 | 2.67805E-74 |
|  | [ENSDARG00000043531](https://www.ensembl.org/id/ENSDARG00000043531) | *jun* | 1.29299004 | 2.51175E-71 |
|  | [ENSDARG00000067850](https://www.ensembl.org/id/ENSDARG00000067850) | *jund* | 1.001413165 | 2.17446E-25 |
|  | [ENSDARG00000074378](https://www.ensembl.org/id/ENSDARG00000074378) | *junba* | 3.014769972 | 9.8318E-138 |
|  | [ENSDARG00000104773](https://www.ensembl.org/id/ENSDARG00000104773) | *junbb* | 3.004885489 | 1.575E-129 |
|  | [ENSDARG00000007823](https://www.ensembl.org/id/ENSDARG00000007823) | *atf3* | 4.173152669 | 2.2058E-245 |
|  | [ENSDARG00000042577](https://www.ensembl.org/id/ENSDARG00000042577) | *batf3* | 3.637578579 | 0.000193563 |
|  | [ENSDARG00000040623](https://www.ensembl.org/id/ENSDARG00000040623) | *fosl2* | 2.086215592 | 4.82381E-71 |
|  | [ENSDARG00000031683](https://www.ensembl.org/id/ENSDARG00000031683) | *fosab* | 3.512134826 | 9.9257E-106 |
|  | [ENSDARG00000015355](https://www.ensembl.org/id/ENSDARG00000015355) | *fosl1a* | 4.793606483 | 1.7807E-108 |
|  | [ENSDARG00000105261](https://www.ensembl.org/id/ENSDARG00000105261) | *nfkb1* | 1.367260037 | 1.3767E-22 |
|  | [ENSDARG00000038687](https://www.ensembl.org/id/ENSDARG00000038687) | *nfkb2* | 2.695596426 | 9.6927E-266 |
|  | [ENSDARG00000005481](https://www.ensembl.org/id/ENSDARG00000005481) | *nfkbiaa* | 4.196678526 | 4.3116E-282 |
|  | [ENSDARG00000007693](https://www.ensembl.org/id/ENSDARG00000007693) | *nfkbiab* | 2.832700179 | 6.8093E-153 |
|  | [ENSDARG00000000857](https://www.ensembl.org/id/ENSDARG00000000857) | *mapk14a* | 0.690899246 | 4.99596E-09 |
|  | [ENSDARG00000054968](https://www.ensembl.org/id/ENSDARG00000054968) | *cd40* | 2.854926279 | 1.26887E-37 |
| Apoptosis | [ENSDARG00000068367](https://www.ensembl.org/id/ENSDARG00000068367) | *nfkbie* | 2.880760277 | 2.0185E-128 |
|  | [ENSDARG00000007693](https://www.ensembl.org/id/ENSDARG00000007693) | *nfkbiab* | 2.832700179 | 6.8093E-153 |
|  | [ENSDARG00000105261](https://www.ensembl.org/id/ENSDARG00000105261) | *nfkb1* | 1.367260037 | 1.3767E-22 |
|  | [ENSDARG00000018569](https://www.ensembl.org/id/ENSDARG00000018569) | *tnfrsf1a* | 1.137108374 | 9.58524E-40 |
|  | [ENSDARG00000089307](https://www.ensembl.org/id/ENSDARG00000089307) | *pmaip1* | 2.563441817 | 8.59623E-47 |
|  | [ENSDARG00000058325](https://www.ensembl.org/id/ENSDARG00000058325) | *casp8* | 1.076933698 | 2.84217E-08 |
|  | [ENSDARG00000004325](https://www.ensembl.org/id/ENSDARG00000004325) | *casp9* | 0.669737816 | 8.33302E-06 |
|  | [ENSDARG00000044125](https://www.ensembl.org/id/ENSDARG00000044125) | *txn* | 0.611697988 | 1.13629E-06 |
|  | [ENSDARG00000043586](https://www.ensembl.org/id/ENSDARG00000043586) | *fas* | 1.998604398 | 1.56869E-07 |
|  | [ENSDARG00000013598](https://www.ensembl.org/id/ENSDARG00000013598) | *tnfb* | 6.458530571 | 4.79822E-59 |
|  | [ENSDARG00000018569](https://www.ensembl.org/id/ENSDARG00000018569) | *tnfrsf1a* | 1.137108374 | 9.58524E-40 |
|  | [ENSDARG00000070165](https://www.ensembl.org/id/ENSDARG00000070165) | *tnfrsf1b* | 0.623271678 | 0.001985681 |
|  | [ENSDARG00000069482](https://www.ensembl.org/id/ENSDARG00000069482) | *traf1* | 2.351034407 | 1.0122E-06 |
|  | [ENSDARG00000022000](https://www.ensembl.org/id/ENSDARG00000022000) | *traf3* | 2.450080497 | 2.7401E-110 |
|  | [ENSDARG00000103021](https://www.ensembl.org/id/ENSDARG00000103021) | *fadd* | 0.687502559 | 0.003254416 |
|  | [ENSDARG00000055966](https://www.ensembl.org/id/ENSDARG00000055966) | *cflara* | 1.564244285 | 4.69026E-33 |
|  | [ENSDARG00000030087](https://www.ensembl.org/id/ENSDARG00000030087) | *nfkbib* | 1.898020492 | 4.5818E-159 |
|  | [ENSDARG00000104172](https://www.ensembl.org/id/ENSDARG00000104172) | *diabloa* | -1.29610401 | 5.45796E-13 |
| Metabolic pathways | [ENSDARG00000099960](https://www.ensembl.org/id/ENSDARG00000099960) | *elovl1a* | 1.143614295 | 7.19292E-19 |
|  | [ENSDARG00000002634](https://www.ensembl.org/id/ENSDARG00000002634) | *b4galt1* | 0.920288321 | 4.5222E-10 |
|  | [ENSDARG00000068515](https://www.ensembl.org/id/ENSDARG00000068515) | *chs1* | 1.147306783 | 2.6792E-08 |
|  | [ENSDARG00000037873](https://www.ensembl.org/id/ENSDARG00000037873) | *cyp3c3_1* | 1.848614069 | 7.98708E-05 |
|  | [ENSDARG00000033999](https://www.ensembl.org/id/ENSDARG00000033999) | *cyp26a1* | 0.840748735 | 1.90396E-10 |
|  | [ENSDARG00000056029](https://www.ensembl.org/id/ENSDARG00000056029) | *cyp26c1* | 0.624970461 | 7.66938E-06 |
|  | [ENSDARG00000092660](https://www.ensembl.org/id/ENSDARG00000092660) | *cyp27c1* | 0.906622072 | 4.19071E-09 |
|  | [ENSDARG00000044002](https://www.ensembl.org/id/ENSDARG00000044002) | *cyp2x7* | 0.738265351 | 5.37279E-06 |
|  | [ENSDARG00000013721](https://www.ensembl.org/id/ENSDARG00000013721) | *g6pca.2* | -0.597861895 | 0.000222731 |
| IFN pathway | [ENSDARG00000040332](https://www.ensembl.org/id/ENSDARG00000040332) | *crfb2* | 0.671752302 | 0.044088656 |
|  | [ENSDARG00000006266](https://www.ensembl.org/id/ENSDARG00000006266) | *stat1a* | 0.968598519 | 7.47768E-18 |
|  | [ENSDARG00000022712](https://www.ensembl.org/id/ENSDARG00000022712) | *stat3* | 0.871161739 | 3.30728E-26 |
|  | [ENSDARG00000028731](https://www.ensembl.org/id/ENSDARG00000028731) | *stat4* | 1.40415886 | 2.86281E-27 |
|  | [ENSDARG00000040465](https://www.ensembl.org/id/ENSDARG00000040465) | *irf2* | 1.16878394 | 1.32179E-15 |
|  | [ENSDARG00000076251](https://www.ensembl.org/id/ENSDARG00000076251) | *irf3* | 2.116991595 | 5.6729E-08 |
|  | [ENSDARG00000045661](https://www.ensembl.org/id/ENSDARG00000045661) | *irf7* | 3.429934573 | 2.67805E-74 |
|  | [ENSDARG00000056407](https://www.ensembl.org/id/ENSDARG00000056407) | *irf8* | 2.91585594 | 6.55059E-39 |
|  | [ENSDARG00000016457](https://www.ensembl.org/id/ENSDARG00000016457) | *irf9* | 1.754093609 | 6.55427E-41 |
|  | [ENSDARG00000027658](https://www.ensembl.org/id/ENSDARG00000027658) | *irf10* | 2.45508554 | 2.41989E-09 |
|  | [ENSDARG00000100515](https://www.ensembl.org/id/ENSDARG00000100515) | *usp18* | 1.356808038 | 3.71939E-33 |
|  | [ENSDARG00000024789](https://www.ensembl.org/id/ENSDARG00000024789) | *mxc* | 4.686314093 | 6.72E-222 |
|  | [ENSDARG00000014427](https://www.ensembl.org/id/ENSDARG00000014427) | *mxe* | 3.487068935 | 9.90318E-10 |
|  | [ENSDARG00000026049](https://www.ensembl.org/id/ENSDARG00000026049) | *mxf* | 7.911131115 | 5.9425E-21 |
|  | [ENSDARG00000052396](https://www.ensembl.org/id/ENSDARG00000052396) | *pkz* | 3.440095787 | 1.99392E-14 |
|  | [ENSDARG00000075643](https://www.ensembl.org/id/ENSDARG00000075643) | *ifi35* | 1.563550641 | 1.8232E-10 |
|  | [ENSDARG00000033227](https://www.ensembl.org/id/ENSDARG00000033227) | *lect2l* | 2.539225385 | 6.07193E-59 |
|  | [ENSDARG00000053136](https://www.ensembl.org/id/ENSDARG00000053136) | *b2m* | 2.277812518 | 6.85911E-19 |
|  | [ENSDARG00000033144](https://www.ensembl.org/id/ENSDARG00000033144) | *psme2* | 1.413848531 | 6.60375E-10 |
|  | [ENSDARG00000075963](https://www.ensembl.org/id/ENSDARG00000075963) | *mhc1uba* | 1.397958741 | 0.001668833 |
|  | [ENSDARG00000055278](https://www.ensembl.org/id/ENSDARG00000055278) | *cfb* | 0.972224152 | 8.18641E-13 |
|  | [ENSDARG00000057121](https://www.ensembl.org/id/ENSDARG00000057121) | *c7b* | 4.052995692 | 1.0642E-162 |
|  | [ENSDARG00000012694](https://www.ensembl.org/id/ENSDARG00000012694) | *c3a.1* | 1.856833498 | 2.77364E-35 |
|  | [ENSDARG00000043719](https://www.ensembl.org/id/ENSDARG00000043719) | *c3a.6* | 1.710418611 | 1.43564E-27 |
| Cytokine-cytokine receptor interaction | [ENSDARG00000058389](https://www.ensembl.org/id/ENSDARG00000058389) | *ccl19a.1* | 2.556672559 | 7.79384E-17 |
|  | [ENSDARG00000039351](https://www.ensembl.org/id/ENSDARG00000039351) | *ccl19b* | 4.454152591 | 1.15614E-60 |
|  | [ENSDARG00000094511](https://www.ensembl.org/id/ENSDARG00000094511) | *ccl20b* | 2.178610749 | 0.022697222 |
|  | [ENSDARG00000101040](https://www.ensembl.org/id/ENSDARG00000101040) | *ccl20a.3* | 7.364631603 | 8.34654E-39 |
|  | [ENSDARG00000090873](https://www.ensembl.org/id/ENSDARG00000090873) | *ccl34a.4* | 3.748738373 | 1.3274E-34 |
|  | [ENSDARG00000103466](https://www.ensembl.org/id/ENSDARG00000103466) | *ccl35.1* | 5.49778464 | 3.23632E-12 |
|  | [ENSDARG00000041917](https://www.ensembl.org/id/ENSDARG00000041917) | *ccl38a.4* | 7.284601668 | 0.02771148 |
|  | [ENSDARG00000104795](https://www.ensembl.org/id/ENSDARG00000104795) | *cxcl8a* | 4.933866107 | 9.5874E-187 |
|  | [ENSDARG00000102299](https://www.ensembl.org/id/ENSDARG00000102299) | *cxcl8b.1* | 1.45532894 | 0.004612333 |
|  | [ENSDARG00000100662](https://www.ensembl.org/id/ENSDARG00000100662) | *cxcl11.1* | 7.381268869 | 0.024550315 |
|  | [ENSDARG00000075045](https://www.ensembl.org/id/ENSDARG00000075045) | *cxcl18b* | 7.888858046 | 0.000426023 |
|  | [ENSDARG00000075163](https://www.ensembl.org/id/ENSDARG00000075163) | *cxcl20* | 1.60561436 | 2.71017E-11 |
|  | [ENSDARG00000070669](https://www.ensembl.org/id/ENSDARG00000070669) | *cxcr3.3* | 2.895604987 | 7.0514E-114 |
|  | [ENSDARG00000022000](https://www.ensembl.org/id/ENSDARG00000022000) | *traf3* | 2.450080497 | 2.7401E-110 |
|  | [ENSDARG00000077207](https://www.ensembl.org/id/ENSDARG00000077207) | *lzts2b* | 0.789865316 | 0.005178829 |
|  | [ENSDARG00000098700](https://www.ensembl.org/id/ENSDARG00000098700) | *il1b* | 7.767926806 | 1.32612E-87 |
|  | [ENSDARG00000104693](https://www.ensembl.org/id/ENSDARG00000104693) | *il6st* | 1.073439398 | 5.60699E-23 |
|  | [ENSDARG00000054542](https://www.ensembl.org/id/ENSDARG00000054542) | *il12bb* | 2.801135414 | 0.012328787 |
|  | [ENSDARG00000057180](https://www.ensembl.org/id/ENSDARG00000057180) | *il17c* | 7.657516422 | 0.0173599 |
|  | [ENSDARG00000089383](https://www.ensembl.org/id/ENSDARG00000089383) | *il1fma* | 2.811768587 | 1.76067E-05 |
|  | [ENSDARG00000102583](https://www.ensembl.org/id/ENSDARG00000102583) | *il4r.1* | 1.780034701 | 4.22616E-34 |
|  | [ENSDARG00000116052](https://www.ensembl.org/id/ENSDARG00000116052) | *il4r.2* | 0.984059225 | 0.000537751 |
|  | [ENSDARG00000074850](https://www.ensembl.org/id/ENSDARG00000074850) | *il12rb2l* | 1.639548161 | 3.88916E-21 |
|  | [ENSDARG00000058244](https://www.ensembl.org/id/ENSDARG00000058244) | *il17ra1a* | 1.119596207 | 4.57141E-15 |
|  | [ENSDARG00000009511](https://www.ensembl.org/id/ENSDARG00000009511) | *tnfa* | 8.489885842 | 0.005531637 |
|  | [ENSDARG00000013598](https://www.ensembl.org/id/ENSDARG00000013598) | *tnfb* | 6.458530571 | 4.79822E-59 |
|  | [ENSDARG00000028731](https://www.ensembl.org/id/ENSDARG00000028731) | *stat4* | 1.40415886 | 2.86281E-27 |
|  | [ENSDARG00000102211](https://www.ensembl.org/id/ENSDARG00000102211) | *csf3a* | 3.817326111 | 2.18863E-12 |
|  | [ENSDARG00000098752](https://www.ensembl.org/id/ENSDARG00000098752) | *csf3b* | 9.931271327 | 0.000540294 |
|  | [ENSDARG00000039863](https://www.ensembl.org/id/ENSDARG00000039863) | *lifrb* | 1.20325555 | 1.76609E-15 |
|  | [ENSDARG00000053624](https://www.ensembl.org/id/ENSDARG00000053624) | *csf1rb* | -2.643535936 | 3.47827E-06 |
|  | [ENSDARG00000038541](https://www.ensembl.org/id/ENSDARG00000038541) | *ccr12a* | -3.196871911 | 0.00081134 |
|  | [ENSDARG00000057633](https://www.ensembl.org/id/ENSDARG00000057633) | *cxcr4a* | -0.829298842 | 0.00529321 |
|  | [ENSDARG00000103208](https://www.ensembl.org/id/ENSDARG00000103208) | *il17ra1b* | 1.145271032 | 0.035555188 |
| Phagosome | [ENSDARG00000018283](https://www.ensembl.org/id/ENSDARG00000018283) | *cyba* | 0.67771808 | 1.71815E-05 |
|  | [ENSDARG00000056615](https://www.ensembl.org/id/ENSDARG00000056615) | *cybb* | 1.128572 | 1.34126E-08 |
|  | [ENSDARG00000006353](https://www.ensembl.org/id/ENSDARG00000006353) | *itga5* | 0.797199272 | 4.21465E-10 |
|  | [ENSDARG00000033735](https://www.ensembl.org/id/ENSDARG00000033735) | *ncf1* | 4.022357866 | 2.4432E-125 |
|  | [ENSDARG00000060410](https://www.ensembl.org/id/ENSDARG00000060410) | *thbs2a* | 0.592372662 | 0.007764054 |
|  | [ENSDARG00000079766](https://www.ensembl.org/id/ENSDARG00000079766) | *tap1* | 2.951289835 | 2.70392E-30 |
|  | [ENSDARG00000036787](https://www.ensembl.org/id/ENSDARG00000036787) | *tap2a* | 3.864415534 | 2.0712E-08 |
|  | [ENSDARG00000033446](https://www.ensembl.org/id/ENSDARG00000033446) | *tap2t* | 1.165757332 | 7.37834E-10 |
|  | [ENSDARG00000016939](https://www.ensembl.org/id/ENSDARG00000016939) | *itgb2* | -1.149808634 | 1.73183E-07 |
|  | [ENSDARG00000005821](https://www.ensembl.org/id/ENSDARG00000005821) | *ncf2* | 1.352878954 | 0.001023026 |
|  | [ENSDARG00000054610](https://www.ensembl.org/id/ENSDARG00000054610) | *coro1a* | -0.791286701 | 0.000535129 |
|  | [ENSDARG00000101794](https://www.ensembl.org/id/ENSDARG00000101794) | *atp6v0e1* | -0.359927509 | 0.021538769 |
|  | [ENSDARG00000038822](https://www.ensembl.org/id/ENSDARG00000038822) | *mrc1b* | -0.838139457 | 0.000208 |
| Autophagy regulators | [ENSDARG00000045561](https://www.ensembl.org/id/ENSDARG00000045561) | *dram1* | 2.822024807 | 3.73028E-13 |
|  | [ENSDARG00000002663](https://www.ensembl.org/id/ENSDARG00000002663) | *optn* | 1.333631641 | 8.0337E-58 |
|  | [ENSDARG00000075014](https://www.ensembl.org/id/ENSDARG00000075014) | *sqstm1* | 0.716368162 | 8.03938E-10 |
|  | [ENSDARG00000063311](https://www.ensembl.org/id/ENSDARG00000063311) | *soga3a_1* | -8.470050032 | 0.022454555 |
| Matrix remodeling | [ENSDARG00000042816](https://www.ensembl.org/id/ENSDARG00000042816) | *mmp9* | 4.741359142 | 5.5061E-167 |
|  | [ENSDARG00000114451](https://www.ensembl.org/id/ENSDARG00000114451) | *mmp13a_2* | 3.110140986 | 4.81582E-57 |
|  | [ENSDARG00000045887](https://www.ensembl.org/id/ENSDARG00000045887) | *mmp30* | 0.649984731 | 1.94004E-07 |
|  | [ENSDARG00000001452](https://www.ensembl.org/id/ENSDARG00000001452) | *adam8a* | 0.631933119 | 0.003056645 |
|  | [ENSDARG00000057644](https://www.ensembl.org/id/ENSDARG00000057644) | *adam8b* | 1.286468436 | 2.7185E-09 |
|  | [ENSDARG00000103519](https://www.ensembl.org/id/ENSDARG00000103519) | *adamts1* | 1.039766947 | 4.40507E-13 |
|  | [ENSDARG00000021859](https://www.ensembl.org/id/ENSDARG00000021859) | *erap1b* | 0.970737921 | 0.000808653 |
|  | [ENSDARG00000010146](https://www.ensembl.org/id/ENSDARG00000010146) | *cpa2* | 2.725574624 | 1.97453E-27 |
|  | [ENSDARG00000043722](https://www.ensembl.org/id/ENSDARG00000043722) | *cpa4* | 1.303291329 | 3.07734E-13 |
|  | [ENSDARG00000075261](https://www.ensembl.org/id/ENSDARG00000075261) | *timp2b* | 2.008499631 | 3.62706E-69 |
|  | [ENSDARG00000010423](https://www.ensembl.org/id/ENSDARG00000010423) | *npsn* | -1.221031822 | 5.50534E-19 |
|  | [ENSDARG00000103878](https://www.ensembl.org/id/ENSDARG00000103878) | *anpepla* | 0.475468402 | 0.000506491 |
|  | [ENSDARG00000076270](https://www.ensembl.org/id/ENSDARG00000076270) | *adamts13* | 0.614451662 | 0.005679621 |
|  | [ENSDARG00000041083](https://www.ensembl.org/id/ENSDARG00000041083) | *anpepa* | 0.838207462 | 0.043494697 |
|  | [ENSDARG00000023656](https://www.ensembl.org/id/ENSDARG00000023656) | *he1.1* | -1.108806293 | 0.040023344 |
